# Multimodular type I polyketide synthases in algae evolve by module duplications and displacement of AT domains *in trans*

**DOI:** 10.1186/s12864-015-2222-9

**Published:** 2015-11-26

**Authors:** Ekaterina Shelest, Natalie Heimerl, Maximilian Fichtner, Severin Sasso

**Affiliations:** Research Group Systems Biology/Bioinformatics, Leibniz Institute for Natural Product Research and Infection Biology (HKI), Beutenbergstr. 11a, 07745 Jena, Germany; Institute of General Botany and Plant Physiology, Friedrich Schiller University, Dornburger Str. 159, 07743 Jena, Germany

**Keywords:** Polyketide synthase, Genomics, Microalgae, Macroalgae, Chlorophyta, Haptophytes, Heterokonts, Dinoflagellates, Cyanobacteria, Toxin

## Abstract

**Background:**

Polyketide synthase (PKS) catalyzes the biosynthesis of polyketides, which are structurally and functionally diverse natural products in microorganisms and plants. Here, we have analyzed available full genome sequences of microscopic and macroscopic algae for the presence of type I PKS genes.

**Results:**

Type I PKS genes are present in 15 of 32 analyzed algal species. In chlorophytes, large proteins in the MDa range are predicted in most sequenced species, and PKSs with free-standing acyltransferase domains (*trans*-AT PKSs) predominate. In a phylogenetic tree, PKS sequences from different algal phyla form clades that are distinct from PKSs from other organisms such as non-photosynthetic protists or cyanobacteria. However, intermixing is observed in some cases, for example polyunsaturated fatty acid (PUFA) and glycolipid synthases of various origins. Close relationships between type I PKS modules from different species or between modules within the same multimodular enzyme were identified, suggesting module duplications during evolution of algal PKSs. In contrast to type I PKSs, nonribosomal peptide synthetases (NRPSs) are relatively rare in algae (occurrence in 7 of 32 species).

**Conclusions:**

Our phylogenetic analysis of type I PKSs in algae supports an evolutionary scenario whereby integrated AT domains were displaced to yield *trans*-AT PKSs. Together with module duplications, the displacement of AT domains may constitute a major mechanism of PKS evolution in algae. This study advances our understanding of the diversity of eukaryotic PKSs and their evolutionary trajectories.

**Electronic supplementary material:**

The online version of this article (doi:10.1186/s12864-015-2222-9) contains supplementary material, which is available to authorized users.

## Background

Polyketide synthases (PKSs) occur in many different microorganisms, where they are involved in the biosynthesis of compounds with a variety of structures and functions. Examples of polyketides include the antibiotic erythromycin from the actinomycete *Saccharopolyspora erythraea* or the cholesterol-lowering agent lovastatin from *Aspergillus terreus* [1–3]. PKS creates chemical complexity by a series of biosynthetic cycles that involves the condensation and modification of simple carboxylic acid building blocks. Its mode of operation, similar to fatty acid synthase (FAS), engages different enzymatic functions: acyltransferase (AT) catalyzes the attachment of a substrate to the acyl carrier protein (ACP) of the PKS, ketosynthase (KS) catalyzes the condensation of two substrates, and ketoreductase (KR), dehydratase (DH) and enoylreductase (ER) catalyze the stepwise processing of the polyketide intermediate. In contrast to FAS, the three processing steps catalyzed by KR, DH and ER are optional in PKS, which results in polyketides with keto groups, hydroxy groups and/or double bonds in different locations [1, 2]. While FAS produces fully saturated fatty acids, another enzyme related to PKS producing polyunsaturated fatty acids (PUFAs) was discovered at the beginning of this century [4]. This PUFA synthase represents an alternative to the classic desaturation/elongation pathway that starts from fully saturated fatty acids [5]. PKSs related to PUFA synthase are involved in the biosynthesis of long-chain polyhydroxy alcohols and contribute to the formation of the glycolipid envelope in cyanobacterial heterocysts [6, 7]. These findings illustrate that FAS, PUFA synthase and PKS are mechanistically closely related, and that FAS and PUFA synthase may be viewed as special cases of PKS [3].

**Fig. 1:**
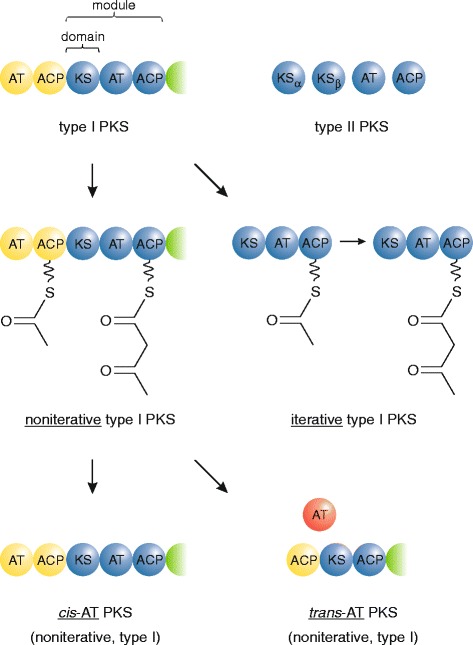
Simplified scheme of PKS subtypes relevant in this work. Type I PKSs (top) are multifunctional proteins, whereas type II PKSs consist of monofunctional proteins that associate into a noncovalent complex. In noniterative type I PKS (middle), which is multimodular, each cycle of elongation and processing is accomplished by one module. In contrast, iterative type I PKS are typically unimodular, and this module is used multiple times during the biosynthesis of a single polyketide. Noniterative type I PKS can have integrated (*cis*) or free-standing (*trans*) AT domains (bottom), whereas basically all iterative type I PKSs feature *cis*-AT domains. See Fig. 2 for domain abbreviations

There are three types of PKS, and two types of FAS. In type I PKS/FAS, the various enzymatic functions are performed by domains within a larger multifunctional protein whereas type II PKS/FAS consists of smaller monofunctional proteins that interact noncovalently [3] (Fig. 1). Type III PKS occurs mainly in land plants and includes chalcone synthase, the enzyme that catalyzes the committed step in flavonoid biosynthesis [8]. In iterative type I PKS, which is typical for fungi, the same set of domains (called a module) is used for each round of polyketide elongation and processing. In contrast, noniterative type I PKS possesses one module for each biosynthetic cycle, which can result in the large multimodular PKSs that are found, for example, in some bacteria [2] (Fig. 1). For the biosynthesis of saturated fatty acids, animals and fungi have different versions of iterative type I FAS, while type II FAS is found in bacteria, mitochondria and plastids [9]. Known FASs and PUFA synthases, involved in the biosynthesis of saturated and unsaturated fatty acids, respectively, are therefore always iterative enzymes. PKS often occurs as a hybrid enzyme with nonribosomal peptide synthetase (NRPS), which resembles noniterative type I PKS, but contains a different set of domains and uses amino acids as building blocks instead of carboxylic acids [1, 10]. For example, the biosynthesis of some cyanobacterial toxins relies on hybrid PKS/NRPS pathways [11].

Algae, an ecologically and biotechnologically important group of aquatic photosynthetic eukaryotes, are phylogenetically diverse [12]. The plastids of the green lineage (chlorophytes, charophytes and land plants), red algae and glaucophytes are derived from a cyanobacterium that was taken up by an ancestral eukaryotic cell during primary endosymbiosis. It is assumed that secondary endosymbiosis of a green alga then led to chlorarachniophytes and euglenozoa, whereas secondary endosymbiosis of a red alga led to heterokonts (which include the diatoms and brown algae), haptophytes, alveolates and cryptophytes. Different species of dinoflagellates, which belong to the alveolates, are the result of either secondary or tertiary endosymbiosis. Genes have been transferred at various times from endosymbiont (plastid) to nucleus in the course of these processes. Therefore, each endosymbiotic event gave rise to a new combination of genes from host and endosymbiont [12], which should be kept in mind during phylogenetic analysis of algal genes. An example is the patchy genome of the haptophyte *Emiliania huxleyi*: Of proteins with orthologs, approximately 30 % showed the highest similarity to proteins from heterokonts (a sister clade of the haptophytes within the red lineage), whereas smaller contributions were inferred from other sources of genetic information such as the green lineage or the eukaryotic ancestor [13].

Since the advent of genome sequencing, the patchy distribution of type I PKS genes in the tree of life has become evident [14–16]. Various mechanisms have been invoked to explain this pattern including gene duplication, gene loss, and horizontal gene transfer [14, 17]. In algae, type I PKS genes were discovered by genome sequencing [15, 18]. At the metabolic level, isotope labeling has demonstrated that toxins and related compounds from several bloom-forming dinoflagellates are made by PKSs [19]. However, no genomes of toxic dinoflagellates have been sequenced. Even though several candidate genes for the biosynthesis of these toxins have been isolated, it has not been possible to establish unambiguous links between specific genes and toxins in dinoflagellates so far [18, 20].

Here, we provide an overview of predicted type I PKS and NRPS genes in sequenced algal genomes, which extends our previous analysis [18]. In addition, the structure and phylogeny of predicted type I PKSs are analyzed in depth. Together, our results provide new insights into PKS diversity, and they culminate in a model of how complex type I PKSs have evolved in algae.

## Results

### Distribution of type I polyketide synthases and nonribosomal peptide synthetases predicted in algae

To refine our picture of the occurrence of type I PKSs and NRPSs in algae, we analyzed genome sequences available for 32 different algal species (Additional file 1: Table S1). For species with several sequenced strains, one representative strain was selected. PKS and NRPS candidates were identified by scanning the genomes for characteristic KS and condensation (C) domains, by BLAST [21] and/or InterProScan [22]. After defining the domain structure of PKS candidates, we filtered out all free-standing KS proteins (as candidates for type II PKSs) and considered all proteins with additional typical PKS domains other than KS (AT, ACP, KR, ER, DH) as type I PKSs (see Methods for more details).

**Fig. 2:**
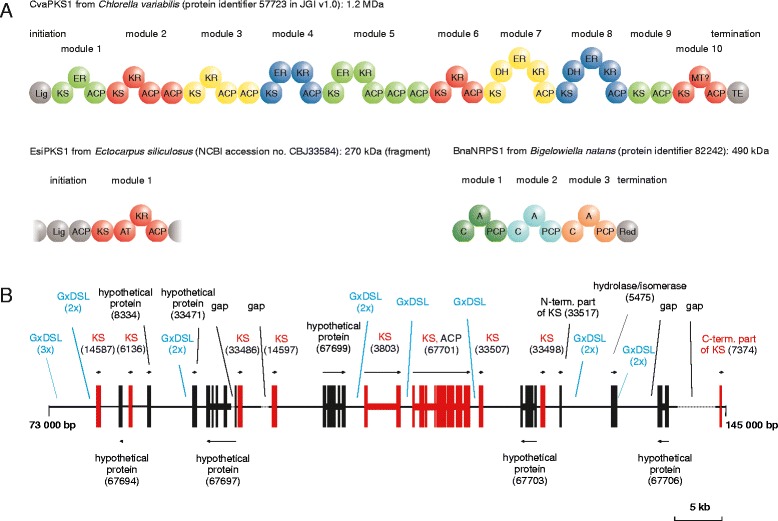
PKSs and NRPSs in algae. **a** Examples of type I PKSs and NRPSs predicted from algal genome sequences. Note that in *Chlorella variabilis*, separate genes are annotated for the N-terminal Lig and KS domains (identifiers 7741 and 22890, respectively), but we assumed that they are part of the large type I PKS gene (57723). EsiPKS1 may be truncated as both ends of the EsiPKS1 gene are close to the ends of the contig. **b** Section of scaffold 46 of the *Aureococcus anophagefferens* genome (position 73,000 - 145,000). Shown are gene models according to the gene prediction in v.1 of the genome together with the corresponding protein identifiers. Wide bars depict exons, narrow bars depict introns. Also shown are sites that encode the conserved GxDSL motif of ACP domains, which we found 15 times in forward direction, but not in reverse direction. Domain abbreviations: KS, ketosynthase; AT, acyltransferase; DH, dehydratase; ER, enoylreductase; KR, ketoreductase; ACP, acyl carrier protein; MT, methyltransferase; Lig, AMP-dependent synthetase/ligase; TE, thioesterase; C, condensation domain; A, adenylation domain; PCP, peptidyl carrier protein; Red, reducing domain

Type I PKSs are quite common in algae but their distribution is uneven: of 32 species examined, 15 possess type I PKSs (Table 1). They seem to be typical for Chlorophyta where they are detected in 9 of 12 species, whereas outside this phylum type I PKSs are only found in a few species distributed over three phyla (Haptophyta, Heterokontophyta, and Dinophyta). So far, all sequenced rhodophytes seem to lack type I PKSs and NRPSs completely (Table 1). Interestingly, one of the species with the highest number of type I PKSs (12 proteins) is *E. huxleyi*, a bloom-forming microalga that belongs to the haptophytes. This is the only haptophyte species that could be included in the study, so it is too early to draw any conclusions, but this observation suggests a high potential for polyketide production in haptophytes. The other species with a large number of PKSs is the chlorophyte *Coccomyxa subellipsoidea* (strain C-169, formerly designated *Coccomyxa* sp. C-169), which is less surprising given the high occurrence of PKSs in Chlorophyta. The large number of PKSs in *C. subellipsoidea* can be most likely explained by the duplication of one or several ancestor genes (we will return to this issue below). In the genome of the brown alga *Ectocarpus siliculosus*, a type I FAS gene is annotated on scaffold 520 [23]. Both ends of the gene model are near the ends of the contig, resulting in a protein that is likely incomplete (Fig. 2a). Nevertheless, the multimodular organisation of this protein fragment suggests that the protein is in reality not a type I FAS, which are generally iterative [24], but a type I PKS. This would be the first example of a type I PKS in a macroalga.

**Table 1:** Occurrence of type I PKSs and NRPSs predicted in algae (zeros omitted to improve legibility)

Species	No. of type I PKSs	No. of KS domains within type I PKSs^a)^	No. of free-standing (type II) KS proteins	No. of NRPSs	No. of C domains within NRPSs^a)^
Streptophyta					
*Klebsormidium flaccidum*			3		
Chlorophyta					
*Asterochloris* sp. Cgr/DA1pho			3		
*Bathycoccus prasinos*			3		
*Chlamydomonas reinhardtii*	1	11 (11)	4		
*Chlorella variabilis*	2	19 (10, 9)	4	1	1 (1)
*Coccomyxa subellipsoidea*	10	19 (10, 9x1)	3	1	1 (1)
*Micromonas* sp. CCMP1545	4	23 (10, 5, 4, 4)	4		
*Micromonas* sp. RCC299	2^b)^	14 (9, 5)	4		
*Monoraphidium neglectum*	8^c)^	13 (3, 2, 2, 2, 1, 1, 1, 1)	28		
*Ostreococcus lucimarinus*	3^d)^	22 (14, 4, 4)	5		
*Ostreococcus tauri*	6	29 (9, 5, 4, 4, 4, 3)	5		
*Ostreococcus* sp. RCC809			2		
*Volvox carteri*	1	11 (11)	3		
Rhodophyta					
*Chondrus crispus*			3		
*Cyanidioschyzon merolae*			2		
*Galdieria sulphuraria*					
*Porphyridium purpureum*					
*Pyropia (Porphyra) yezoensis*			2		
Glaucophyta					
*Cyanophora paradoxa*				1^e)^	2 (2)^e)^
Chlorarachniophyta					
*Bigelowiella natans*			4	1	3 (3)
Heterokontophyta					
*Aureococcus anophagefferens*	1^f)^	1 (1)	56^f)^	1	3 (3)
*Ectocarpus siliculosus*	1	1 (1)	5		
*Fragilariopsis cylindrus*			3		
*Nannochloropsis gaditana* B-31	2	2 (1, 1)	3		
*Nannochloropsis oceanica* CCMP1779	4	4 (4x1)	7		
*Phaeodactylum tricornutum*			2		
*Pseudo-nitzschia multiseries*			3		
*Thalassiosira oceanica*				1	1 (1)
*Thalassiosira pseudonana*			4		
Dinophyta					
*Symbiodinium minutum* ^g)^	17	n.s.	n.s.	6	n.s.
Haptophyta					
*Emiliania huxleyi*	12^h)^	60 (13, 13, 7, 5, 4, 4, 3, 3, 3, 3, 1, 1)	8		
Cryptophyta					
*Guillardia theta*			6		

^a)^Numbers in brackets indicate the numbers of KS or C domains within the different predicted type I PKS or NRPS proteins, respectively

^b)^MicPKS2 may contain N-terminal C and A domains, but the C domain has a slightly deviating HHxxxD motif (HHIVCE)

^c)^Many contigs in *M. neglectum* are small so that most likely, some genes can be merged. Therefore, this number is only a rough approximation

^d)^OluPKS3 may contain a central C domain (including HHxxxD motif), but no A domain is predicted

^e)^The NRPS gene in *C. paradoxa* is incomplete (gap at 5'-end)

^f)^In reality, a significant number of proteins annotated as freestanding KS proteins in *A. anophagefferens* are most likely part of type I PKSs, which would increase the number of type I PKSs and decrease the number of free-standing KSs (see main text for more details)

^g)^The numbers for *S. minutum* are highly approximate estimates and refer to the ~40% of the 1.5-Gb genome that is sequenced and assembled (see main text for more details). Many genes may be truncated because of the small size of the contigs. One potential hybrid PKS-NRPS gene (identifier 12436) was counted as a PKS. Due to high uncertainties, data on individual KS and C domains are not shown (n.s.)

^h)^EhuPKS1 and EhuPKS2 may each contain a single C domain (including HHxxxD motif), but no A domains are predicted

As stated previously [18], many of the predicted microalgal type I PKSs are very large proteins. For example, the genome of *Chlorella variabilis* encodes two type I PKSs, each with a subunit size of 1.2 MDa. Demonstrated for bacterial and fungal type I PKSs [3, 25], homodimerization is also expected to occur in algae. In CvaPKS1 from *C. variabilis*, a subunit consists of approximately 40 domains arranged into 12 modules (Fig. 2a). Alternatively, it is possible that the predicted homodimer subunit is in reality translated as multiple polypeptide chains that interact non-covalently, in a fashion similar to, for example, erythromycin biosynthesis in the bacterium *S. erythraea* [26].

Unlike type I PKSs, NRPSs are rare in algae. Nevertheless, they are predicted to occur in 7 of 32 species examined, with a total of 11 C domains distributed over 6 proteins (Table 1); more C domains are found in numerous NRPSs of *Symbiodinium minutum*, but the numbers are preliminary (see next section). NRPSs are therefore much less frequent in eukaryotic microalgae than in cyanobacteria, where they occur in more than half of species as self-contained NRPS or hybrid NRPS/PKS systems [16]. The largest microalgal NRPS known so far exists in *Bigelowiella natans*, with a predicted subunit size of 490 kDa and a total of 10 domains (Fig. 2a). A protein that shares the same domain arrangement is encoded by the genome of *Aureococcus anophagefferens* (subunit size 460 kDa).

In summary, the distribution of type I PKSs and NRPSs is phylogenetically scattered in algae, suggesting a large potential for the production of polyketides and nonribosomal peptides in some lineages, whereas others completely lack this ability. In almost all cases, the function of algal type I PKSs and NRPSs and the structure of their respective polyketide and nonribosomal peptide products remains mysterious (see Discussion).

### Challenges in quality of genome assembly and annotation

In a phylogenetic tree constructed for all available KS domains, the great majority of free-standing (type II) KSs do not mix with KSs from type I PKSs, but form separate clades. In some cases however, we noticed that proteins annotated as free-standing KSs are found within type I PKS clades (not shown). Strikingly, groups of these KSs are encoded by clusters of co-directional genes. In the case of *C. variabilis*, re-annotation of the genomic regions containing these genes (see Methods) revealed one additional multidomain protein with a size of 1.2 MDa (CvaPKS2) (Additional file 2: Table S2 and Additional file 3). In *Micromonas* sp. CCMP1545, we found that a gene fragment could be merged with an adjacent gene to yield a larger gene that encodes MccPKS1. Hints from phylogenetic analysis thus helped us to improve gene prediction and annotation of these type I PKSs that had been wrongly annotated (fully or partly) as sets of type II genes, and to identify one new type I PKS in *C. variabilis*.

In *A. anophagefferens*, a unicellular heterokont known to form toxic brown tides in estuaries [27], re-annotation is less straightforward. The published annotation of its genome sequence contains many individual genes for type II PKS/FAS [27]. The total number of KS domains in *A. anophagefferens* is among the largest in all considered algae (Table 1). The majority of the more than 50 predicted KS proteins are encoded by clusters of co-directional genes. For example, scaffolds 40 and 46 contain several co-directional KS genes [27]. A section of scaffold 46 is shown in Fig. 2b. To investigate whether this scaffold has coding potential for additional PKS proteins/domains, we looked for stretches that encode the conserved GxDSL motif of ACP domains (thereby avoiding the complex problem of gene prediction). The same strand of scaffold 46 that encodes KS proteins/domains was found to encode the GxDSL motif several times, whereas no GxDSL-encoding stretches were found on the opposite strand (Fig. 2b).

Interestingly, the recently released version 3.0 of antiSMASH (a program optimized for bacteria and fungi) [28] detected several distinct genes for both KS and adenylation (A) domains (the latter part of NRPSs) on scaffold 46, while earlier versions did not yield any hits. Again, all predicted A domains have the same orientation as the KS domains (not shown). Together, these results suggest that due to incorrect gene prediction, only the highly conserved KS-encoding regions were originally annotated in *A. anophagefferens*, and many type I PKS genes may have evaded detection. As re-annotation is difficult without additional information such as expression data, KSs from *A. anophagefferens* are counted as free-standing proteins in Table 1.

*S. minutum*, belonging to a genus of dinoflagellates symbiotic with marine invertebrates, has a very large genome of approximately 1500 Mb [29]. In a remarkable effort, Shoguchi et al. have sequenced and assembled 600 Mb of sequence (~40 %) into ~22,000 scaffolds [29]. Some PKSs and NRPSs may be truncated due to the small size of many scaffolds (L_50_ = 0.13 Mb). In addition to the enormous genome size and the resulting incomplete assembly, spliced leader (SL)-mediated *trans*-splicing of primary transcripts is a feature that complicates the analysis of the *S. minutum* genome sequence. *Trans*-splicing is abundant in dinoflagellates [30] and affects at least 20 % of genes in *S. minutum* [29]. As a consequence, it may not be easy to identify a genomic region that encodes a distinct protein. For all these reasons, we have not attempted a detailed analysis of type I PKS and NRPS genes in *S. minutum*. Nevertheless, it is clear that type I PKS genes are abundant in this dinoflagellate, which also possesses several NRPS and possibly hybrid PKS-NRPS genes ([29], and data not shown). It is interesting to note that even though the *S. minutum* culture used for genome sequencing contained bacteria, the majority of PKS and NRPS genes possess introns [29], providing strong evidence that these genes indeed belong to *S. minutum*.

### Module and domain architecture of algal type I polyketide synthases

It was suggested that the noniterative assembly line mechanism, which is often found in bacteria, is absent from most eukaryotic organisms because it is costly [31]. It however seems that microalgae and possibly even macroalgae, as primary producers, are able to afford such large genes and thus represent an exception from this rule: many algal type I PKSs comprise multiple modules, hence their mode of catalysis is expected to be noniterative. Iterative (unimodular) type I PKSs are found in less than half of all species possessing type I PKSs (Additional file 2: Table S2). Interestingly, of all unimodular proteins, only those from *C. subellipsoidea* (CsuPKS2, CsuPKS3, and CsuPKS5 to CsuPKS9) have a classical domain structure (KS-AT-MT-ER-KR-ACP) and are in this sense reminiscent of fungal iterative enzymes.

A striking feature of most algal noniterative type I PKSs is the lack of an integrated AT domain (Fig. 2a, Additional file 2: Table S2). Indeed, almost all of the ~30 noniterative PKSs described in this work rely on an AT domain provided *in trans. trans*-AT domains (Fig. 1) are iteratively used to acylate each module [32]. Our data suggest that in algae, *cis*-AT domains are characteristic for iterative (unimodular) PKSs, and vice versa, noniterative (multimodular) PKSs generally lack integrated AT domains. Accordingly, the presence of an integrated AT domain in the seven proteins from *C. subellipsoidea* mentioned above matches the regular architecture of iterative type I PKS [33] and is thus in agreement with our assignment of these proteins as iterative PKSs. In addition, these seven PKSs, but also CsuPKS1 from the same alga, possess a *C*-methyltransferase (MT) domain (Additional file 2: Table S2). In type I PKSs, such MT domains are often employed by noniterative *trans*-AT and by iterative enzymes to methylate the growing polyketide chain at the α-position, whereas noniterative *cis*-AT enzymes achieve the same modification by using methylmalonyl-CoA in place of malonyl-CoA as extender unit [32, 34]. The seven iterative type I PKSs from *C. subellipsoidea* are examples of such MT-containing enzymes. On the other hand, the complete absence of MT domains seems to be a prominent feature of most algal *trans*-AT PKSs. Possibly, free-standing MT domains may be employed by some algal *trans*-AT PKSs, as has been suggested, for example, in the case of oxazolomycin biosynthesis in *Streptomyces* [32].

**Fig. 3:**
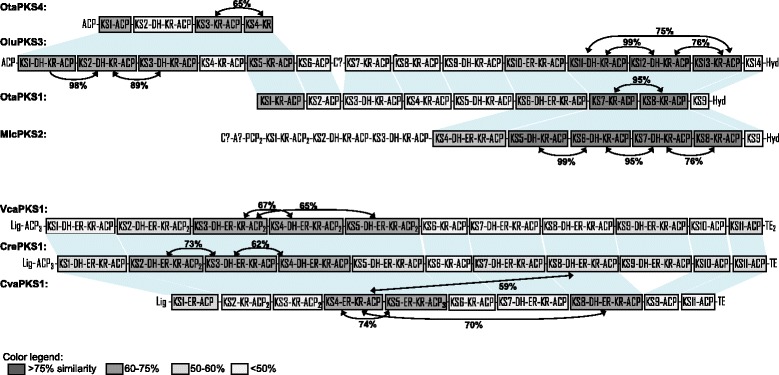
Examples of module duplication in type I PKSs from algae. Module sequences were compared at the protein level by bidirectional BLAST. The degree of similarity between the modules is reflected by the color code. For the most striking examples of module similarity the percentage of identity is specified. Blue-shaded connections between proteins: similarity suggested by the phylogenetic analysis. See legend to Fig. 2 for domain abbreviations and a comment on the structure of the enzyme from *Chlorella variabilis*. Abbreviations: Cre, *Chlamydomonas reinhardtii*; Cva, *Chlorella variabilis*; Mic, *Micromonas* sp. RCC299; Olu, *Ostreococcus lucimarinus*; Ota, *Ostreococcus tauri*; Vca, *Volvox carteri*

At the level of modules, high sequence similarity can be detected in several cases, both between modules of a single PKS, and between modules of different PKSs from the same or different algae. For example, both OtaPKS1 and OtaPKS4 from *Ostreococcus tauri* share highly similar motifs with OluPKS3 from *Ostreococcus lucimarinus* (Fig. 3). Moreover, the overall structure of OtaPKS4 from *O. tauri* is very similar to the first five modules of OluPKS3 (indicated by blue-shaded connections in Fig. 3). Interestingly, modules 1 to 3 of OluPKS3 represent a nice example of apparent module duplication: the overall similarity between the modules ranges from 89 to 98 %, the KS and ACP domains being conserved to 98-100 %. Another striking example of duplications conserved through several species comprises the modules 11 to 13 of OluPKS3, modules 7 and 8 of OtaPKS1, and modules 5 to 8 of MicPKS2 from *O. lucimarinus*, *O. tauri*, and *Micromonas* sp. RCC299, respectively. These modules are highly conserved within their respective PKSs, but also strongly correspond to each other in the three orthologous PKSs. Module duplications can also be observed in several other species such as *C. variabilis* (Fig. 3) or *C. subellipsoidea* (not shown). Additionally, the seven iterative PKSs from *C. subellipsoidea* (CsuPKS2, -3, and -5 to -9) are most likely the product of a whole-gene duplication: the mutual similarity between them ranges from 53 to 95 %. Four of these genes (encoding CsuPKS3, -7, -8, -9) lie in close proximity on the chromosome [18] and are 91-95 % identical, so they likely result from a recent tandem duplication.

In addition, we can also observe domain duplications within one module, even though these events are less numerous than module duplications. For instance, in *Volvox carteri* VcaPKS1 an apparent recent duplication of ACP domains can be seen in module 5 (data not shown). Thus, the picture of the evolution of multimodular PKSs in Chlorophyta looks like a patchwork of domain and module duplications on the framework of highly similar structures, which are homologous in sometimes quite distant species such as *Micromonas* and *Ostreococcus*.

### Phylogenetic tree and evolution of microalgal type I polyketide synthases

**Fig. 4:**
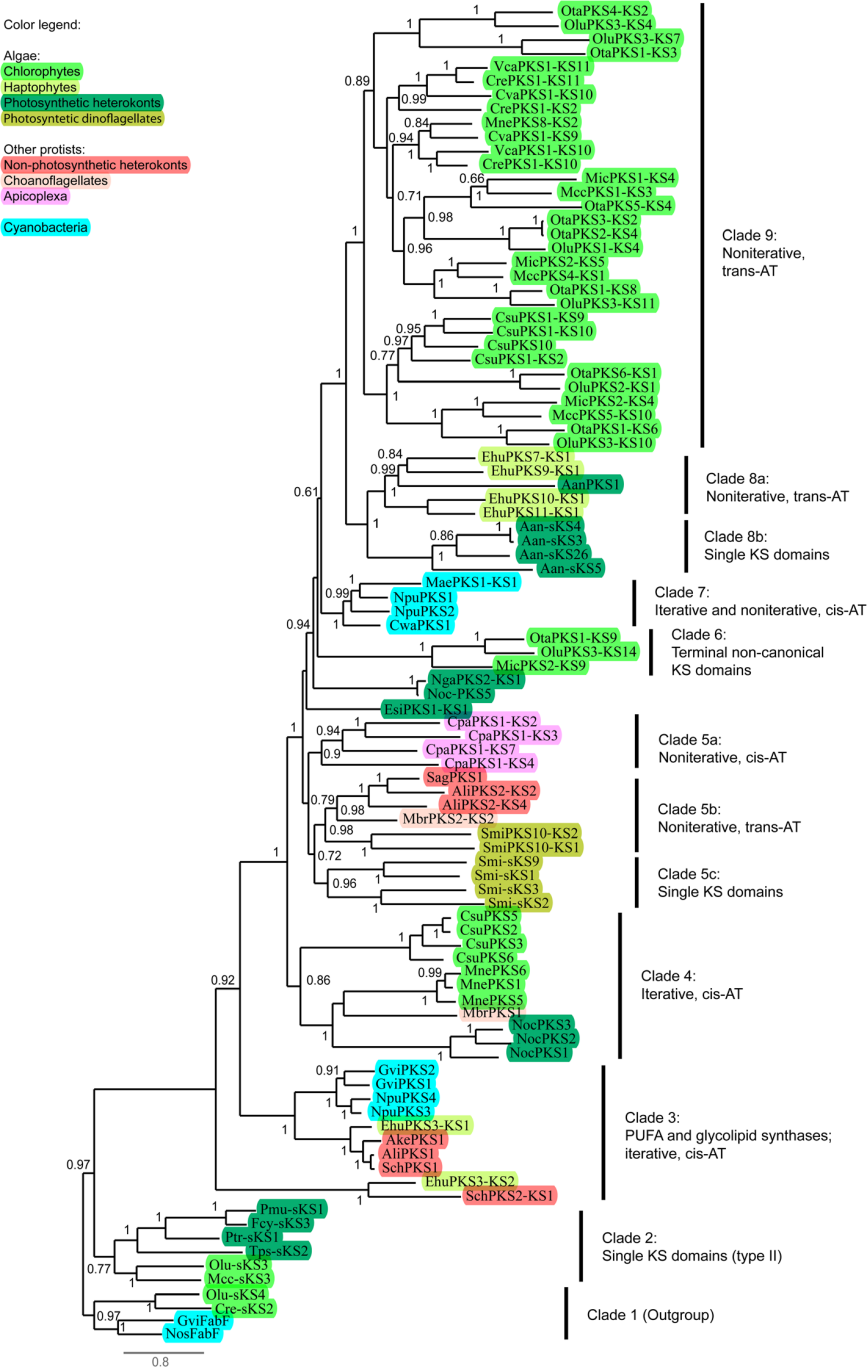
Phylogenetic tree of KS amino acid sequences from algal PKSs. From an alignment built by MUSCLE, a maximum likelihood (ML) tree was constructed by PhyML using sequences of FabF (involved in type II fatty acid biosynthesis) from cyanobacteria and algae as the outgroup. Branch support values were calculated with aBayes likelihood-based method. The scale bar indicates substitutions per site. Abbreviations: sKS, single (free-standing) KS domains; Ali, *Aurantiochytrium limacinum*; Ake, *Aplanochytrium kerguelense*; Aan, *Aureococcus anophagefferens*; Cpa, *Cryptosporidium parvum*; Cre, *Chlamydomonas reinhardtii*; Csu, *Coccomyxa subellipsoidea*; Cva, *Chlorella variabilis*; Cwa, *Crocosphaera watsonii*; Ehu, *Emiliania huxleyi*; Esi, *Ectocarpus siliculosus*; Fcy, *Fragilariopsis cylindrus*; Gvi, *Gloeobacter violaceus*; Mae, *Microcystis aeruginosa*; Mbr, *Monosiga brevicolis*; Mcc, *Micromonas* sp. CCMP1545; Mic, *Micromonas* sp. RCC299; Mne, *Monoraphidium neglectum*; Nga, *Nannochloropsis gaditana*; Noc, *Nannochloropsis oceanica*; Nos, *Nostoc* sp. PCC 7120; Npu, *Nostoc punctiforme*; Olu, *Ostreococcus lucimarinus*; Ota, *Ostreococcus tauri*; Pmu, *Pseudo-nitzschia multiseries*; Ptr, *Phaeodactylum tricornutum*; Sag, *Schizochytrium aggregatum*; Sch, *Schizochytrium* sp. ATCC 20888; Smi, *Symbiodinium minutum*; Tps, *Thalassiosira pseudonana*; Vca, *Volvox carteri*

To obtain new insights into the phylogenetic relationship of algal type I PKSs and their evolutionary history, we constructed phylogenetic trees with the sequences of representative KS domains from sequenced algal species (Fig. 4). For comparison and to get a better overview, we also included in the analysis selected sequences from other unicellular eukaryotes and cyanobacteria. The robustness of the obtained tree is reflected by the high supports for the clades and practically identical tree architectures obtained by different methods (Additional file 1: Figure S1, and Methods). As expected, algal and cyanobacterial FabF sequences that were used as the outgroup clustered together at the base of the tree (clade 1 in Fig. 4). Together with clade 2, clade 1 contains a large number of algal type II KS sequences; for clarity, only some are shown in Fig. 4. Adjacent to clade 2 is a clade of KS sequences (clade 3) that are part of iterative enzymes from various cyanobacteria and eukaryotes. The enzymes in clade 3 have been implicated in glycolipid biosynthesis in the case of cyanobacteria, or in PUFA biosynthesis in the case of the remaining organisms (e.g., [4, 14]; see Additional file 2: Table S2 for additional information). All these verified and predicted glycolipid and PUFA synthases have AT domains *in cis*.

Clades 4 and 5 contain sequences from protists that are both photosynthetic (algae) and non-photosynthetic. Like clade 3, clade 4 contains iterative type I PKSs with *cis*-AT domains. It is interesting to note that iterative PKSs from chlorophytes in clade 4 are distant from noniterative PKSs from the same algal phylum (clade 9). Similarly, photosynthetic heterokonts contribute to separate clades: While clade 4 includes iterative PKSs from *Nannochloropsis oceanica*, clade 8 contains sequences from *A. anophagefferens* that phylogenetically resemble noniterative PKSs from the haptophyte *E. huxleyi*, including Aan-sKS5 that is encoded on scaffold 46 (annotated as free-standing KS). This finding further supports the idea that *A. anophagefferens* contains several type I PKSs. Similar to the clear separation of iterative and noniterative PKSs from algae observed here, iterative and noniterative PKSs from bacteria and fungi also form separate clades [31].

PKSs from the dinoflagellate *S. minutum* are found in clade 5. Interestingly, the free-standing (single) KS domains from *S. minutum* form a distinct subclade (5c), which is very close to type I PKS domains from the same species. As in the case of *A. anophagefferens*, all these KS proteins may be part of type I PKSs in reality. Cyanobacterial PKSs are found in clade 7, including McyD (designated MaePKS1 in Fig. 4), a two-module PKS from *Microcystis aeruginosa* involved in the biosynthesis of the toxin microcystin [11]. Clades 6, 8 and 9 contain KS domains solely from algal PKSs (Fig. 4). In the chlorophyte clades 6 and 9, sequences from different species of this phylum intermix, even though sequences from the taxonomic class of Chlorophyceae (*M. neglectum*, *V. carteri*, *Chlamydomonas reinhardtii*) generally cluster with each other and with sequences from *C. variabilis* (Trebouxiophyceae), and sequences from the class of Prasinophyceae (*Micromonas* sp. RCC299, *O. lucimarinus*, *O. tauri*) cluster with each other. Clade 8a is formed by the haptophyte *E. huxleyi* and includes KS domains from all PKSs of this species (for the sake of space, only some are shown) except for EhuPKS3, which clusters with PUFA/glycolipid synthases in clade 3.

The small clade 6 contains terminal KS domains of three type I PKSs from chlorophytes (MicPKS2, OluPKS3, OtaPKS1). In all three cases, mutations in the HGTGT consensus motif include a change of the conserved active-site histidine required for decarboxylative condensation [35] to serine (Additional file 1: Table S3). Therefore, these KS domains are expected to be non-elongating (so-called KS^0^ domains). In addition, the conserved cysteine required for transacylation is shifted forward by two positions within the DTACSSS consensus motif (e.g., to DCASASG in OluPKS3). Within their respective PKS, the KS^0^ domains from clade 6 are located in front of a hydrolase-like domain (InterPro family IPR027417) and may thus be involved in the release of the polyketide product, or in its transfer to another PKS subunit. In other terminal KS domains of type I PKSs from chlorophytes, the identity and position of the conserved cysteine and histidine residues are retained, but changes at the second and third positions of the HGTGT motif are notable. For example, this motif is altered to HANGT in the type I PKSs from *C. reinhardtii* and *V. carteri* (in terminal KS domains CrePKS1-KS11 and VcaPKS1-KS11, respectively). In contrast to the KS^0^ domains from clade 6, CrePKS1-KS11 and VcaPKS1-KS11 mix well with other canonical KS domains in the phylogenetic tree (Fig. 4).

To increase the resolution of *trans*-AT PKSs from Chlorophyceae, Trebouxiophyceae and Prasinophyceae in the phylogenetic tree, additional trees were constructed using only sequences from these three classes (Additional file 1: Figure S2). In several cases, KS domains from different species intermix, reflecting the similarity between the modules of homologous PKSs. For example, the mutual correspondence of module 9 of CvaPKS1, module 10 of CrePKS1, and module 10 of VcaPKS1 (Fig. 3) is reflected by the clustering of the respective KS domains into one clade (Additional file 1: Figure S2A). Similarly, MicPKS2-KS4 clusters with OluPKS3-KS10 and OtaPKS1-KS6, and the domains of OtaPKS5 correspond to the domains of MicPKS1 (Additional file 1: Figure S2B) even though the ancestors of *Micromonas* and *Ostreococcus* diverged more than 300 million years ago [36].

## Discussion

### Algal type I polyketide synthases probably cover a variety of functions

The present study analyzed the abundance and evolution of type I PKSs and NRPSs encoded by algal genomes. Type I PKSs were found in approximately half of algal species (Table 1). We are not aware of any experimental data on polyketides in sequenced algae, apart from evidence that building blocks of phlorotannins are produced by a type III PKS in *E. siliculosus* [37]. Therefore, the function of the large majority of type I PKSs is unknown to date. Only in *E. huxleyi*, one of the 12 predicted type I PKSs, EhuPKS3, forms a clade with glycolipid and PUFA synthases, whereas other PKSs in *E. huxleyi* are located in another part (clade 8a) of the phylogenetic tree (Fig. 4). The hypothesis that EhuPKS3 is involved in the biosynthesis of PUFAs or polyhydroxy alcohols (such as those that are part of cyanobacterial glycolipids) is further supported by its domain arrangement ([18] and Additional file 2: Table S2).

In basically all other cases, type I PKSs may not act as FAS or PUFA synthase in the production of standard saturated or unsaturated fatty acids: Most algal type I PKSs are noniterative, and their domain arrangement does not match the usual organisation [4, 24] of known FASs and PUFA synthases. At the same time, algae in general have a type II FAS in the plastid [24], and unsaturated fatty acids are usually derived from saturated fatty acids via an oxygen-dependent desaturation/elongation pathway [5]. While some PKSs may be involved in toxin biosynthesis, in the harmful bloom former *A. anophagefferens*, no distinct toxins have been identified in this species [27]. On the other hand, *E. huxleyi* can cause massive blooms [13]. In contrast to some other haptophytes such as *Prymnesium parvum* [38], blooms of *E. huxleyi* may not pose any harm, and no toxins have been reported from this species. Similarly, blooms of *Ostreococcus* are not believed to harm marine animals [39], and all chlorophytes investigated in this work are generally considered harmless. Therefore, the function of most algal type I PKSs remains enigmatic. Similarly, the function of algal NRPSs remains unclear, and no experimental studies have been published yet. Given the structural diversity of these genes, it is likely that even orthologs will be responsible for distinct products, and we can expect a range of functions in different species.

### Evolution of algal type I polyketide synthases

**Fig. 5:**
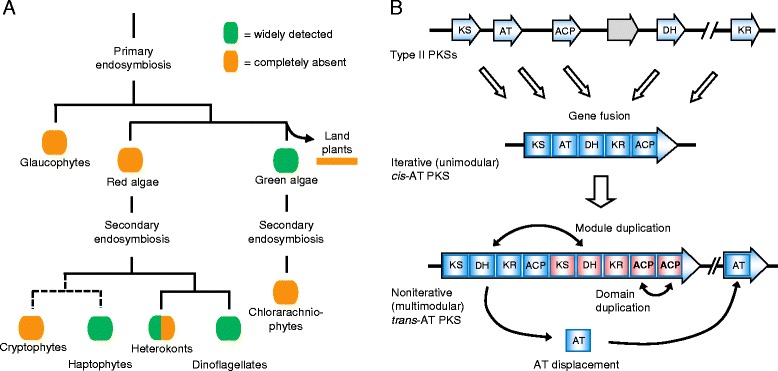
PKS evolution in algae. **a** Occurrence of PKS in different groups of algae. The scheme of endosymbiotic history was adopted from ref. [12]. **b** Working model for the molecular evolution of algal PKSs. Like bacterial type I PKSs, algal type I PKS evolved by the fusion of ancestral type II genes, which first gave rise to iterative (unimodular) type I PKS. Subsequently, AT domains were displaced to be encoded by separate genes, partially reversing the previous fusion step. Duplications of modules and domains then resulted in noniterative (multimodular) type I PKS. Information on the order of AT displacements and duplications is currently lacking (see Discussion for details). Note that early steps of this scenario may have occurred in bacteria

In general, KS sequences from protists (including microalgae) and bacteria form phylogenetically distinct clades (Fig. 4), as observed in previous analyses [15, 40, 41]. This distinct protistan clade argues against a recent lateral transfer from bacteria or fungi [15]. The close relationship between PKSs from the haptophyte *E. huxleyi* (clade 8a) and those from chlorophytes (clades 6 and 9) (Fig. 4) was noted by John and co-workers as well [15]. At the same time, we also see some intermixing in cases of specific classes of type I PKS, particularly in clade 3 (iterative glycolipid and PUFA synthases). This clade includes synthases from a heterotrophic bacterium (*Shewanella* sp.) (not shown), cyanobacteria, non-photosynthetic eukaryotes, and one alga (*E. huxleyi*) (Fig. 4). The patchy occurrence of type I PKSs in algae does not match their endosymbiotic history (Table 1 and Fig. 5a). In the red lineage, the observed pattern suggests multiple acquisitions or multiple losses of type I PKSs, but it is currently not possible to distinguish between these two possibilities. The widespread occurrence of these enzymes in chlorophytes with the simultaneous absence from red algae, streptophytes (including land plants) and possibly chlorarachniophytes (one species analyzed) is even more interesting (Fig. 5a): Chlorophytes may have acquired type I PKSs after their divergence from the streptophytes, which occurred 500 to 1200 million years ago [36], either by independent evolution from algal type II systems or by horizontal gene transfer from bacteria. Again however, differential loss may provide an alternative explanation.

At the molecular level, a popular evolutionary scenario assumes that type I PKS/FAS is derived from type II PKS/FAS by gene fusion, and that bacterial type I PKSs share a common ancestor with animal FAS [14, 42, 43]. Our phylogenetic analysis suggests that algal type I PKSs are also derived from type II systems (Fig. 4). Algal *cis*-AT and *trans*-AT PKSs tend to group separately, in clades 3 and 4, and in clades 5b, 6, 8a and 9, respectively (Fig. 4). Their relative positions suggest AT domains are being displaced from *cis*-AT PKSs over time. A more pronounced formation of distinct clades of *cis*-AT and *trans*-AT PKSs, independent of species, has been observed in bacteria and interpreted as evidence for an independent evolution of *cis*-AT and *trans*-AT PKSs [44]. In contrast, our phylogenetic analysis indicates that the evolution of *cis*- and *trans*-ATs in algae is unambiguously connected to the evolution of multimodularity. Photosynthetic heterokonts, which are distant from both Chlorophyta and Haptophyta [45], have iterative PKSs with integrated AT domains, with the exception of *A. anophagefferens*, which may possess noniterative PKSs. (The pecularities and uncertainties about *A. anophagefferens* PKSs have been discussed above.) The haptophyte *E. huxleyi* generally follows the rule that algal noniterative PKSs depend on free-standing AT domains: of 12 PKSs, 9 are noniterative, and 8 of these lack an integrated AT domain. Only the noniterative EhuPKS9 may contain a single AT domain *in cis*. All iterative type I PKSs (EhuPKS4, EhuPKS12 and PUFA/glycolipid synthase EhuPKS3) possess *cis*-AT domains. With the exception of iterative PKSs from *C. subellipsoidea* (CsuPKS2, -3, -5 to -9) and *M. neglectum* (MnePKS1, -5 and -6), all chlorophyte PKSs are noniterative with AT domains *in trans*.

Pairwise comparison of PKS sequences (Fig. 3) and phylogenetic analysis (Fig. 4, Additional file 1: Figure S2) indicated that module and whole-gene duplications constitute a pivotal evolutionary mechanism. For example, we see evidence for the duplication of some KS domains, which is in fact a reflection of the duplication of the whole module. As discussed above, several modules within one PKS in, for example, *Ostreococcus* are nearly identical (Fig. 3), which suggests that they are the product of duplication; these duplications are supported by the phylogenetic tree (reflected by close relationships between domains: e.g., MicPKS2-KS5 to -KS8, or OluPKS3-KS1 to -KS3 and -KS5; Additional file 1: Figure S2B).

Remarkable examples of high sequence similarity, indicative of module duplication within and between genes, have also been observed in bacteria [43]. Nonetheless, algal PKSs may not necessarily evolve in the same way as bacterial PKSs. Our findings point to several events of genetic duplication in different algal species, including examples of recent duplications, and suggest that this mechanism plays a particular role in the evolution of algal PKS genes. Integrating the important evolutionary mechanisms of the displacement of AT domains (Fig. 4) with gene duplication and fusion, we can propose a model of the molecular evolution of algal PKS (Fig. 5b). According to this working model, type II genes fused at an early stage to form genes for unimodular (iterative) type I PKSs with *cis*-AT domains. Displacement of the AT domain *in trans* and duplication of modules and domains then led to noniterative *trans*-AT PKSs that are found in many algae. The order of these two steps is currently unclear: While it seems more logical that AT displacement preceded module duplications, there is little convincing evidence for the existence of iterative *trans*-AT PKSs (Additional file 2: Table S2), which is the necessary intermediate in this process. For some unknown reason, this evolutionary intermediate might be very short-lived. On the other hand, there are several examples of noniterative *cis*-AT PKSs (Additional file 2: Table S2), which would support a route where module duplications preceded AT displacement. In one alternative scenario (not shown), fusion of type II genes may have resulted in both iterative *cis*-AT PKSs and iterative *trans*-AT PKSs (evolved in parallel), and the latter may then have evolved into noniterative *trans*-AT PKSs. However, the apparent absence of *trans*-AT PKSs disfavors this alternative scenario.

## Conclusions

By analyzing available genome sequences, this work demonstrates the widespread occurrence of type I PKSs in algae. A striking abundance of large noniterative PKSs with free-standing AT domains is found in chlorophytes. In addition, phylogenetic analysis helped to identify several cases where type I PKS genes appeared misannotated as multiple type II genes. Finally, gene duplication and displacement of AT domains are implicated as important mechanisms of PKS evolution in algae, with some duplications having occurred quite recently.

## Methods

### Data retrieval, domain analysis and extraction, and re-annotation

Algal genome information was obtained from the Joint Genome Institute (JGI) and other resources listed in Additional file 1: Table S1. Putative PKS and NRPS candidates were selected by scanning the predicted protein models for typical PKS and NRPS domains by BLAST [21] and/or InterProScan [22], depending on the availability of the databases. BLASTP searches were performed with standard parameters (E-value set to 1e-4) using as query amino acid sequences of the first KS domain (KS1) of *E. huxleyi* EhuPKS1 (JGI ID 631889) and *Micromonas* sp. RCC299 MicPKS1 (JGI ID 55049), and of the first C domain (C1) of the NRPS from *A. anophagefferens* (JGI protein ID 70689). InterProScan genome-wide predictions were scanned for IPR014030 (PF00109) and IPR014031 (PF02801) for the N- and C-terminal portions of the KS domain, respectively, and for IPR001242 (PF00668) for the C domain. Proteins containing KS or C domains were retrieved and the prediction of the other domains and the overall domain structure were then refined by InterProScan, PKS-NRPS analysis tool [46], antiSMASH 2.0 [47], and manual inspection. Because some domains of algal PKSs are rarely detected by bioinformatics tools, DH and MT domains were predicted if the respective conserved motifs HxxxGxxxxP and (D/E)xGxGxG [3] were present in appropriate regions of the protein. Proteins were considered as type I PKS if they contained at least one KS domain that includes the conserved cysteine residue within a DTACSSS motif and a histidine within a HGTGT motif, and at least one additional typical PKS domain (ACP, AT, ER, KR, DH). All proteins that contained the minimal set of NRPS domains (A, C, PCP) and at least one C domain with a conserved HHxxxD motif were considered as NRPSs.

The start and stop positions of the KS domains were denoted by InterProScan. The predictions by N- and C-terminal-specific HMMs were merged if the distance between the domain moieties was not larger than half of the typical domain length (350 aa). The domains were extracted using Java scripts and used for the construction of the phylogenetic trees. All KS domains were checked for the DTACSSS and HGTGT consensus motifs, and all C domains for the HHxxxD motif.

For re-annotation in two cases, genomic regions from *C. variabilis* and *Micromonas* sp. CCMP1545 were submitted to AUGUSTUS [48] trained for different closely and distantly related species (*C. reinhardtii*, *Arabidopsis thaliana*, *Galdieria sulphuraria*, *Solanum lycopersicum*), yielding CvaPKS2 and MccPKS1.

### Alignments and phylogenetic analyses

A total of 302 KS domains were subjected to a phylogenetic analysis, however, the trees were made from subsets: a selection of non-redundant sequences for the main tree and two sets of Chlorophyta KSs for additional trees. For the main tree, the set was cleaned of redundant (identical or nearly identical) domains from the same protein for the sake of tree size. Alignments were performed by MUSCLE [49, 50] and ClustalW [51] with Gonnet protein weight matrix and otherwise standard parameters. Both alignments were used in parallel for the tree reconstruction by two methods, maximum likelihood (ML) and neighbor joining (NJ). The best evolutionary model for the ML analysis was selected by the ProtTest [52]. For all trees, the best model according to AIC (Akaike Information Criteria) was Le-Gascuel with empirical frequencies, estimated proportion of invariable sites, and estimated gamma shape parameter. ML trees were built by PhyML v3.0.1 [53], and statistical branch supports were computed with aBayes likelihood-based method. NJ trees were built using PHYLIP [54] with Jones-Taylor-Thornton model, the bootstrap analysis for the supports was performed with 1000 replicates.

A consensus tree for the four trees obtained (MUSCLE/PhyML, MUSCLE/NJ, Clustal/PhyML, Clustal/NJ) was computed by the Consense Phylip 3.67 package on the Mobyle portal [55]. The architectures of the consensus tree and each single tree were compared by tanglegrams, performed by the EPoS framework for phylogenetic analysis [56]. The architecture of the MUSCLE/PhyML tree (shown in Fig. 4) was in best agreement (practically identical) with that of the consensus tree (Additional file 1: Figure S1). For the Chlorophyta KS trees, the domains were aligned by ClustalX and the trees were built by PhyML with the settings described above.

A second genome sequence of a haptophyte, Chrysochromulina tobin, was published recently [57] . This paper reports several type I PKS and hybrid PKS/NRPS genes, supporting the potential of haptophytes for polyketide biosynthesis.

## Additional files

Additional file 1:
**Table S1.** Overview of available algal genome sequences. **Table S3.** Overview of conserved amino acid motifs in selected algal type I PKSs. **Figure S1.** Comparison of the phylogenetic tree from Fig. 4 (MUSCLE/PhyML) with a consensus tree from trees constructed by four different methods (MUSCLE/PhyML, MUSCLE/NJ, Clustal/PhyML, Clustal/NJ). **Figure S2.** Detailed phylogenetic trees of KS domains from different classes of Chlorophyta (Chlorophyceae, Trebouxiophyceae, Prasinophyceae). Additional references. (DOCX 729 kb) Additional file 2:
**Table S2.** Details on analyzed type I PKSs and NRPSs. (XLSX 22 kb) Additional file 3:
**Amino acid sequences of newly annotated type I PKSs.** (DOCX 25 kb)
