# Supplementary material for: Multimodular type I polyketide synthases in algae evolve by module duplications and displacement of AT domains in trans
Source: BMC Genomics. 2015 Nov 26;16:1015. doi: 10.1186/s12864-015-2222-9 (PMC4661987; doi:10.1186/s12864-015-2222-9)
Supplement: Additional file 1: — Table S1. Overview of available algal genome sequences. Table S3. Overview of conserved amino acid motifs in selected algal type I PKSs. Figure S1. Comparison of the phylogenetic tree from Fig. 4 (MUSCLE/PhyML) with a consensus tree from trees constructed by four different methods (MUSCLE/PhyML, MUSCLE/NJ, Clustal/PhyML, Clustal/NJ). Figure S2. Detailed phylogenetic trees of KS domains from different classes of Chlorophyta (Chlorophyceae, Trebouxiophyceae, Prasinophyceae). Additional references. (DOCX 729 kb) [file 12864_2015_2222_MOESM1_ESM.docx]

**Additional file 1**

**Multimodular type I polyketide synthases in algae evolve**

**by module duplications and displacement of AT domains**

***in trans***

Ekaterina Shelest, Natalie Heimerl, Maximilian Fichtner, Severin Sasso

Contents:

**Table S1** Overview of available algal genome sequences

**Table S3** Overview of conserved amino acid motifs in selected algal type I PKSs

**Figure S1** Comparison of the phylogenetic tree from Fig. 3(MUSCLE/PhyML) with a consensus tree from trees constructed by four different methods (MUSCLE/PhyML, MUSCLE/NJ, Clustal/PhyML, Clustal/NJ)

**Figure S2** Detailed phylogenetic trees of KS domains from different classes of Chlorophyta. (a) Chlorophyceae (*Chlamydomonas reinhardtii, Volvox carteri*) and Trebouxiophyceae (*Chlorella variabilis, Coccomyxa subellipsoidea*). (b) Prasinophyceae (*Micromonas* sp. RCC299, *Ostreococcus lucimarinus, Ostreococcus tauri*).

**Additional references**

**Additional file 1: Table S1** Overview of available algal genome sequences

| Species | Description | Genome size (Mb) | Gene number | Reference | Internet portal or NCBI accession number | Current version |  |
| --- | --- | --- | --- | --- | --- | --- | --- |
| Chlorophyta |  |  |  |  |  |  |  |
| *Asterochloris* sp. Cgr/DA1pho ^1)^ | lichen photobiont | 55.8 | 7,159 | accessible online | <http://genome.jgi-psf.org/Astpho2> | v3 |  |
| *Bathycoccus prasinos* ^1)^ | marine picoeukaryote | 15.1 | 7,847 | [[1](#_ENREF_1)] | <http://bioinformatics.psb.ugent.be/genomes/view/Bathycoccus-prasinos> |  | |
| *Chlamydomonas reinhardtii* ^1)^ | model species, freshwater | 111.1 | 17,741 | [[2](#_ENREF_2)] | <http://phytozome.jgi.doe.gov/pz/portal.html#!info?alias=Org_Creinhardtii> | v5.5 |  |
| *Chlorella* *variabilis* ^1)^ | *Paramecium* symbiont | 46.2 | 9,791 | [[3](#_ENREF_3)] | http://genome.jgi-psf.org/ChlNC64A_1 | v1.0 |  |
| *Coccomyxa subellipsoidea* ^1)^ | *Chlorella* relative | 49 | 9,627 | [[4](#_ENREF_4)] | <http://genome.jgi-psf.org/Coc_C169_1> | v2.0 |  |
| *Micromonas* sp. CCMP1545 ^1)^ | marine picoeukaryote | 21.9 | 10,672 | [[5](#_ENREF_5)] | [http://genome.jgi-psf.org/MicpuC](http://genome.jgi-psf.org/MicpuC2)3 | v3.0 |  |
| *Micromonas* sp. RCC299 ^1)^ | marine picoeukaryote | 21 | 10,109 | [[5](#_ENREF_5)] | <http://genome.jgi-psf.org/MicpuN3> | v3.0 |  |
| *Monoraphidium neglectum* ^1)^ | microalgae with increased lipid production | ca. 68 | 16,761 | [[6](#_ENREF_6)] | AYTC01000000 |  |  |
| *Ostreococcus lucimarinus* ^1)^ | marine picoeukaryote | 13.2 | 7,651 | [[7](#_ENREF_7)] | http://genome.jgi-psf.org/Ost9901_3 | v2.0 |  |
| *Ostreococcus tauri* ^1)^ | marine picoeukaryote | 12.6 | 7,725 | [[8](#_ENREF_8)] | <http://genome.jgi-psf.org/Ostta4> | v2.0 |  |
| *Ostreococcus* sp. RCC809 | marine picoeukaryote | 13.3 | 7,492 | accessible online | <http://genome.jgi-psf.org/OstRCC809_2> | v2.0 |  |
| *Volvox carteri* ^1)^ | simple multicellular relative of *C. reinhardtii* | 131.2 | 14,971 | [[9](#_ENREF_9)] | <http://phytozome.jgi.doe.gov/pz/portal.html#!info?alias=Org_Vcarteri> | v2.0 |  |
|  |  |  |  |  |  |  |  |
| Rhodophyta |  |  |  |  |  |  |  |
| *Chondrus crispus* ^1)^ | multicellular seaweed, source of carrageenan | 105 | 9,606 | [[10](#_ENREF_10)] | http://www.sb-roscoff.fr/en-abiotic-stress-research-topics/1681-the-chondrus-crispus-genome-project.html |  |  |
| *Cyanidioschyzon merolae* ^1)^ | thermo-acidophile | 16.5 | 5,331 | [[11](#_ENREF_11)] | <http://merolae.biol.s.u-tokyo.ac.jp> |  |  |
| *Galdieria sulphuraria* | thermo-acidophile | 13.7 | 6,623 | [[12](#_ENREF_12)] | http://genomics.msu.edu/galdieria |  |  |
| *Porphyridium purpureum* | mesophilic unicellular alga | 19.7 | 8,355 | [[13](#_ENREF_13)] | http://cyanophora.rutgers.edu/porphyridium |  |  |
| *Pyropia (Porphyra) yezoensis* ^1)^ | multicellular seaweed, susabi-nori | ca. 43 | 10,327 | [[14](#_ENREF_14)] | http://nrifs.fra.affrc.go.jp/ResearchCenter/5_AG/genomes/nori |  |  |
|  |  |  |  |  |  |  |  |
| Glaucophyta |  |  |  |  |  |  |  |
| *Cyanophora paradoxa* ^1)^ | freshwater alga with peptidoglycan-surrounded plastid | ca. 70 | 27,921 | [[15](#_ENREF_15)] | http://cyanophora.rutgers.edu/cyanophora |  |  |
| Species | Description | Genome size (Mb) | Gene number | Reference | Internet portal or NCBI accession number | Current version |  |
|  |  |  |  |  |  |  |  |
| Chlorarachniophyta |  |  |  |  |  |  |  |
| *Bigelowiella natans* ^1)^ | marine alga | 94.7 | 21,708 | [[16](#_ENREF_16)] | http://genome.jgi.doe.gov/Bigna1 | v1.0 |  |
|  |  |  |  |  |  |  |  |
| Heterokontophyta |  |  |  |  |  |  |  |
| *Aureococcus anophagefferens* | harmful algal bloom-forming marine pelagophyte | 56.7 | 11,501 | [[17](#_ENREF_17)] | [http://genome.jgi-psf.org/Auran1](http://genome.jgi-psf.org/Auran1/Auran1.home.html) | v1.0 |  |
| *Ectocarpus siliculosus* | brown alga (multicellular seaweed) | 214 | 16,256 | [[18](#_ENREF_18)] | http://bioinformatics.psb.ugent.be/webtools/bogas/overview/Ectsi |  |  |
| *Fragilariopsis cylindrus* ^1)^ | marine psychrophilic diatom | 80.5 | 27,137 | accessible online | <http://genome.jgi-psf.org/Fracy1> | v1.0 |  |
| *Nannochloropsis gaditana* B-31 ^1)^ | marine lipid-accumulating alga | 28.5 | 10,486 | [[19](#_ENREF_19)] | http://www.nannochloropsis.org |  |  |
| *Nannochloropsis gaditana*  CCMP526 ^1)^ | marine lipid-accumulating alga | 29 | 8,892 | [[20](#_ENREF_20)] | http://www.nannochloropsis.org |  |  |
| *Nannochloropsis oceanica*  LAMB0001 | marine lipid-accumulating alga | ca. 30 | 11,129 | [[21](#_ENREF_21)] | AEUM00000000 |  |  |
| *Nannochloropsis oceanica*  CCMP1779 | marine lipid-accumulating alga | 28.7 | 11,973 | [[22](#_ENREF_22)] | https://bmb.natsci.msu.edu/about/directory/faculty/christoph-benning/nannochloropsis-oceanica-ccmp1779 |  |  |
| *Phaeodactylum tricornutum* ^1)^ | marine pennate diatom | 27.4 | 10,402 | [[23](#_ENREF_23)] | [http://genome.jgi-psf.org/Phatr2](http://genome.jgi-psf.org/Phatr2/) | v2.0 |  |
| *Pseudo-nitzschia multiseries* | neurotoxin-producing diatom | 218.7 | 19,703 | accessible online | http://genome.jgi.doe.gov/Psemu1 | v1.0 |  |
| *Thalassiosira oceanica* ^1)^ | oceanic diatom | 81.6 | 29,306 | [[24](#_ENREF_24)] | AGNL00000000 |  |  |
| *Thalassiosira pseudonana* ^1)^ | marine centric diatom | 32.4 | 11,776 | [[25](#_ENREF_25)] | http://genome.jgi-psf.org/Thaps3 | v3.0 |  |
|  |  |  |  |  |  |  |  |
| Dinoflagellates |  |  |  |  |  |  |  |
| *Symbiodinium minutum* ^1)^ | unicellular symbiotic alga (photosynthetic) | ca. 1,500  (616 Mb sequenced) | ca. 42,000 | [[26](#_ENREF_26)] | http://marinegenomics.oist.jp/genomes/viewer?project_id=21&current_assembly_version=symb_aug_v1.120123 | v1.  120123 |  |
|  |  |  |  |  |  |  |  |
| Haptophyta |  |  |  |  |  |  |  |
| *Emiliania huxleyi* | marine coccolithophore | 167.7 | 30,569 | [[27](#_ENREF_27)] | http://genome.jgi-psf.org/Emihu1 | v1.0 |  |
|  |  |  |  |  |  |  |  |
|  |  |  |  |  |  |  |  |
| Species | Description | Genome size (Mb) | Gene number | Reference | Internet portal or NCBI accession number | Current version |  |
|  |  |  |  |  |  |  |  |
| Cryptophyta |  |  |  |  |  |  |  |
| *Guillardia theta*^1)^ | marine alga | 87.2 | 24,840 | [[16](#_ENREF_16)] | http://genome.jgi.doe.gov/Guith1 | v1.0 |  |
| Charophyta |  |  |  |  |  |  |  |
| *Klebsormidium flaccidum*^1)^ | filamentous terrestrial alga | ca. 117.1 | 16,063 | [[28](#_ENREF_28)] | [http://www.plantmorphogenesis.bio.titech.ac.jp/~algae_genome_project/klebsormidium/index.html](http://www.plantmorphogenesis.bio.titech.ac.jp/%7Ealgae_genome_project/klebsormidium/index.html) | v1.0 |  |

^1)^ Genome size and gene number refer to the nuclear genome; in all other cases it was not defined.

**Additional file 1: Table S3** Overview of conserved amino acid motifs in selected algal type I PKSs. Abbreviations are as in Figure 4.

| KS domain | 1st motif | 2nd motif | Comment |
| --- | --- | --- | --- |
| AanPKS2 | DTACSTA | HGTGT |  |
| CpaPKS1-KS2 | DTACSSS | HGTGT |  |
| CpaPKS1-KS3 | DAACASS | HGTGT |  |
| CpaPKS1-KS4 | DTACSSS | HGTGT |  |
| CpaPKS1-KS7 | DTACSSS | HGTGT |  |
| CrePKS1-KS10 | DTACSSS | HGTGT |  |
| CrePKS1-KS11 | DTACSAS | HANGT | Terminal domain |
| CsuPKS10 | DTACSSS | HGTGT |  |
| CsuPKS1-KS10 | DTACSSS | HGTGT |  |
| CsuPKS1-KS2 | DTACSSS | HGTGT |  |
| CsuPKS1-KS9 | DTACSSS | HGTGT |  |
| CsuPKS2 | DTACSSS | HGTGT |  |
| CsuPKS3 | DTACSSS | HGTGT |  |
| CsuPKS5 | DTACSSS | HGTGT |  |
| CsuPKS6 | DTACSSA | HGTGT |  |
| CvaPKS1-KS8 | DTACSSS | HGTGT |  |
| CvaPKS1-KS9 | DTACSAS | HSNGT | Terminal domain |
| CwaPKS1 | DTTCSSS | HGTGT |  |
| EhuPKS10-KS1 | DTACSAA | AANGS |  |
| EhuPKS11-KS1 | DTACSAA | HGTGT |  |
| EhuPKS3-KS1 | DAACASS | HGTGT |  |
| EhuPKS3-KS2 | DAACASA | HATGT |  |
| EhuPKS7-KS1 | DTACSSA | HGTGT |  |
| EhuPKS9-KS1 | DTACSSS | HGTGT |  |
| EsiPKS1 | DTACSSS | HGTGT |  |
| GviPKS1 | DAACASS | HGTGT |  |
| GviPKS2 | DAACASS | HGTGT |  |
| MaePKS1-KS1 | DTACSSS | HGTGT |  |
| MicPKS1-KS4 | DTACSSS | HGTGT |  |
| MicPKS2-KS4 | DTACSSS | HGTGT |  |
| MicPKS2-KS5 | DTACSSS | HGTGT |  |
| MicPKS2-KS9 | DCASASA | SGLGE | Terminal domain |
| NgaPKS3 | DTACSSS | HGTGT |  |
| NocPKS1 | ETACSSS | HATGT |  |
| NocPKS2 | ETACSSS | HATGT |  |
| NocPKS3 | ETACSSS | HATGT |  |
| NocPKS5 | DTACSSS | ----- |  |
| NpuPKS1 | DTACSSS | HGTGT |  |
| NpuPKS2 | DTACSSS | HGTGT |  |
| NpuPKS3 | DAACASS | HGTGT |  |
| NpuPKS4 | DAACASS | HGTGT |  |
| OluPKS1-KS4 | DTACSSS | HGTGT |  |
| OluPKS2-KS1 | DTACSSS | HGTGT |  |
| OluPKS3-KS10 | DTACSSS | HGTGT |  |
| OluPKS3-KS11 | DTACSSS | HGTGT |  |
| OluPKS3-KS14 | DCASASG | SGLGE | Terminal domain |
| OluPKS3-KS4 | DTACSSA | HGTGT |  |
| OluPKS3-KS7 | DTACSSS | HGTGT |  |
| OtaPKS1-KS3 | DTACSSS | HGTAT |  |
| OtaPKS1-KS6 | DTACSSS | HGTGT |  |
| OtaPKS1-KS8 | DTACSSS | HGTGT |  |
| OtaPKS1-KS9 | DCASASG | SGLGE | Terminal domain |
| OtaPKS2-KS4 | DTACSSS | HGTGT |  |
| OtaPKS3-KS2 | DTACSSS | HGTGT |  |
| OtaPKS4-KS2 | DTACSSA | HGTGT |  |
| OtaPKS5-KS4 | DTACSSS | HGTGT |  |
| OtaPKS6-KS1 | DTACSSS | HGTGT |  |
| VcaPKS1-KS10 | DTACSSS | HGTGT |  |
| VcaPKS1-KS11 | DTACSSS | HANGT | Terminal domain |

**Additional file 1: Figure S1** Comparison of the phylogenetic tree from Figure 4 (MUSCLE/PhyML) with a consensus tree from trees constructed by four different methods (MUSCLE/PhyML, MUSCLE/NJ, Clustal/PhyML, Clustal/NJ). See Methods for details.


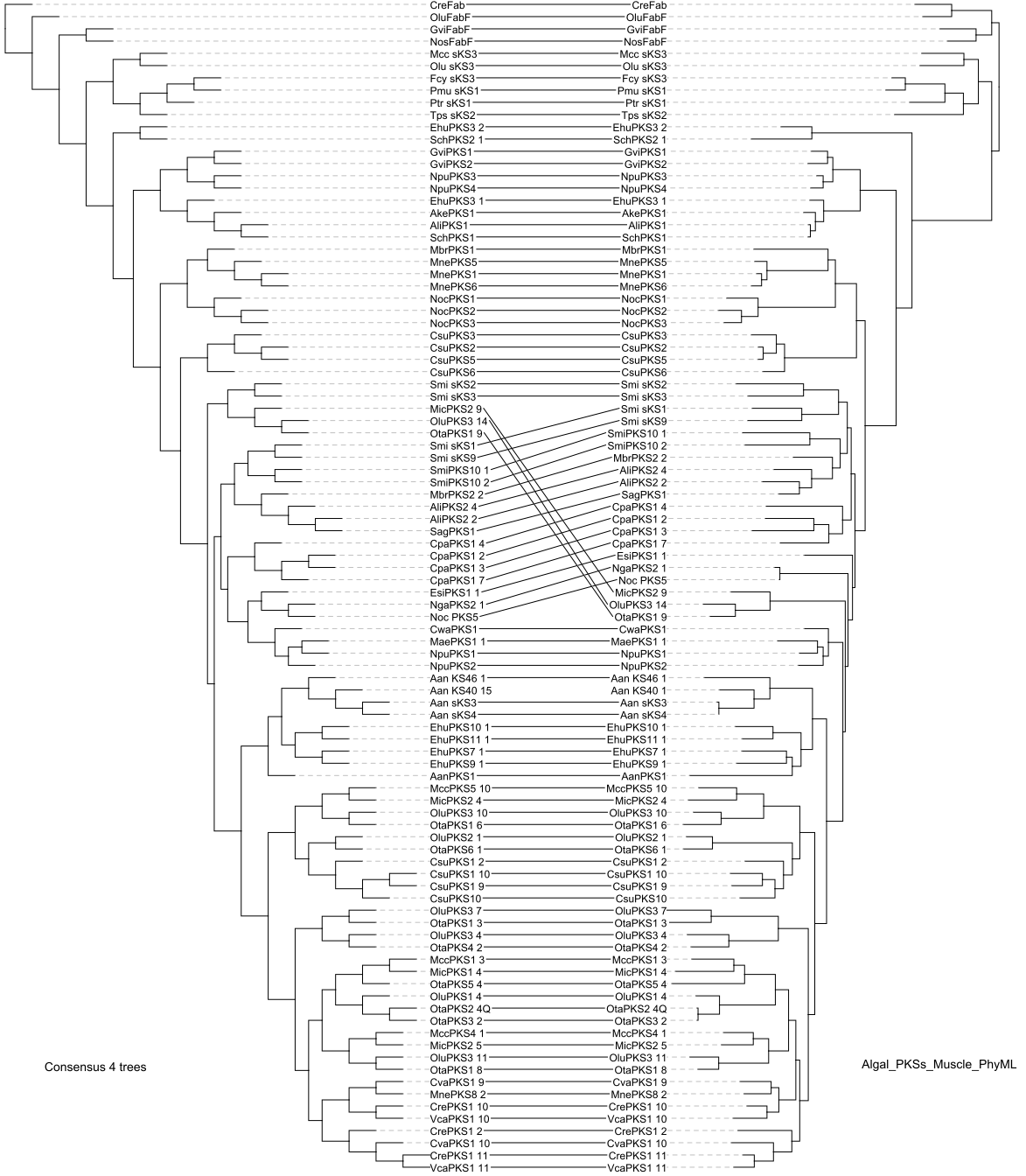


**Additional file 1: Figure S2** Detailed phylogenetic trees of KS domains from different classes of Chlorophyta. (A) Chlorophyceae (*Chlamydomonas reinhardtii*, *Volvox carteri*) and Trebouxiophyceae (*Chlorella variabilis*, *Coccomyxa subellipsoidea*). (B) Prasinophyceae (*Micromonas* sp. RCC299, *Ostreococcus lucimarinus*, *Ostreococcus tauri*). The scale bar indicates substitutions per site.

(A)


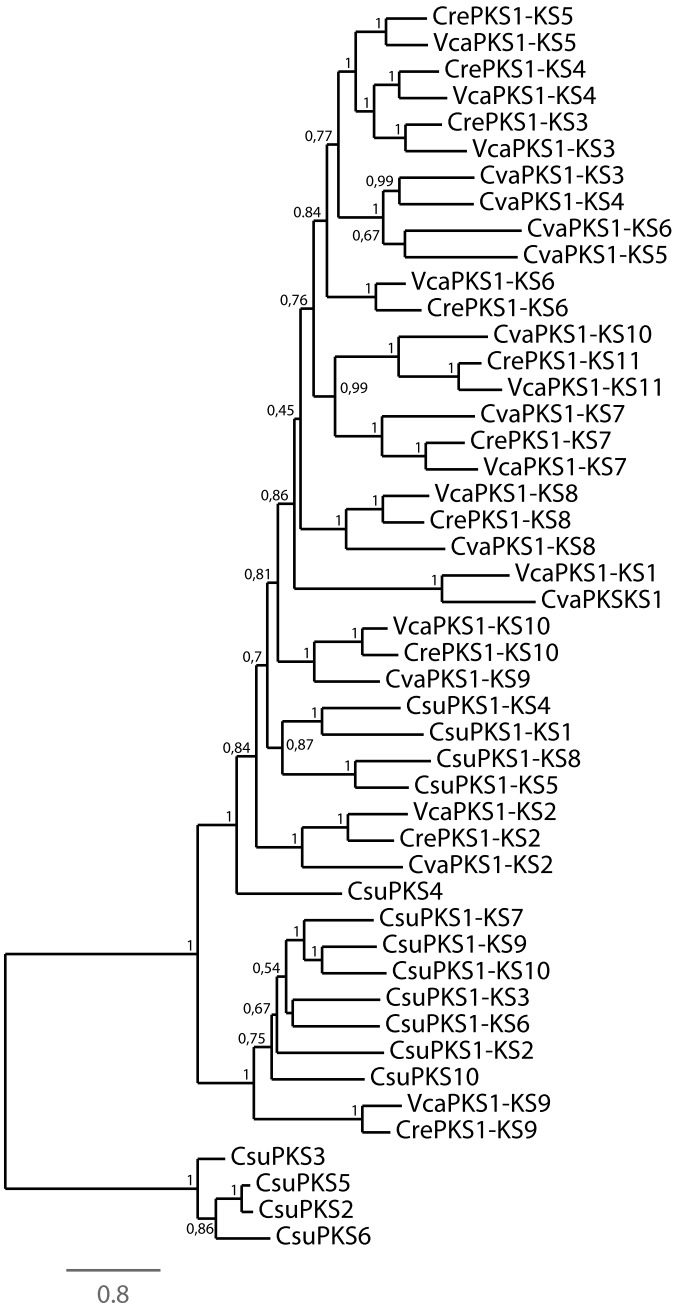


(B)


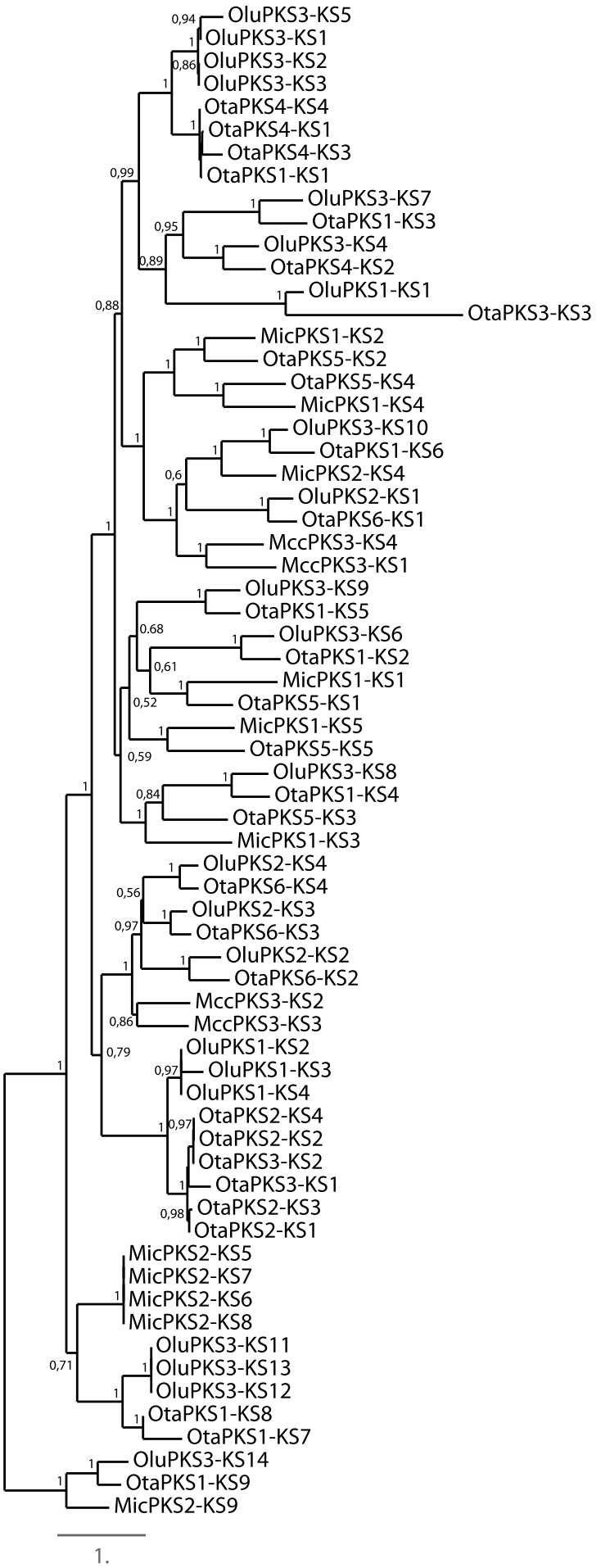


**Additional file 1: Additional references**

1. Moreau H, Verhelst B, Couloux A, Derelle E, Rombauts S, Grimsley N et al. **Gene functionalities and genome structure in *Bathycoccus prasinos* reflect cellular specializations at the base of the green lineage**. *Genome Biol* 2012, **13**:R74.

2. Merchant SS, Prochnik SE, Vallon O, Harris EH, Karpowicz SJ, Witman GB et al. **The *Chlamydomonas* genome reveals the evolution of key animal and plant functions**. *Science* 2007, **318**:245-251.

3. Blanc G, Duncan G, Agarkova I, Borodovsky M, Gurnon J, Kuo A et al. **The *Chlorella variabilis* NC64A genome reveals adaptation to photosymbiosis, coevolution with viruses, and cryptic sex**. *Plant Cell* 2010, **22**:2943-2955.

4. Blanc G, Agarkova I, Grimwood J, Kuo A, Brueggeman A, Dunigan DD et al. **The genome of the polar eukaryotic microalga *Coccomyxa subellipsoidea* reveals traits of cold adaptation**. *Genome Biol* 2012, **13**:R39.

5. Worden AZ, Lee J-H, Mock T, Rouzé P, Simmons MP, Aerts AL et al. **Green evolution and dynamic adaptations revealed by genomes of the marine picoeukaryotes *Micromonas***. *Science* 2009, **324**:268-272.

6. Bogen C, Al-Dilaimi A, Albersmeier A, Wichmann J, Grundmann M, Rupp O et al. **Reconstruction of the lipid metabolism for the microalga *Monoraphidium neglectum* from its genome sequence reveals characteristics suitable for biofuel production**. *BMC Genomics* 2013, **14**:926.

7. Palenik B, Grimwood J, Aerts A, Rouzé P, Salamov A, Putnam N et al. **The tiny eukaryote *Ostreococcus* provides genomic insights into the paradox of plankton speciation**. *Proc Natl Acad Sci U S A* 2007, **104**:7705-7710.

8. Derelle E, Ferraz C, Rombauts S, Rouzé P, Worden AZ, Robbens S et al. **Genome analysis of the smallest free-living eukaryote *Ostreococcus tauri* unveils many unique features**. *Proc Natl Acad Sci U S A* 2006, **103**:11647-11652.

9. Prochnik SE, Umen J, Nedelcu AM, Hallmann A, Miller SM, Nishii I et al. **Genomic analysis of organismal complexity in the multicellular green alga *Volvox carteri***. *Science* 2010, **329**:223-226.

10. Collén J, Porcel B, Carre W, Ball SG, Chaparro C, Tonon T et al. **Genome structure and metabolic features in the red seaweed *Chondrus crispus* shed light on evolution of the Archaeplastida**. *Proc Natl Acad Sci U S A* 2013, **110**:5247-5252.

11. Matsuzaki M, Misumi O, Shin-i T, Maruyama S, Takahara M, Miyagishima S et al. **Genome sequence of the ultrasmall unicellular red alga *Cyanidioschyzon merolae* 10D**. *Nature* 2004, **428**:653-657.

12. Schönknecht G, Chen W-H, Ternes CM, Barbier GG, Shrestha RP, Stanke M et al. **Gene transfer from bacteria and archaea facilitated evolution of an extremophilic eukaryote**. *Science* 2013, **339**:1207-1210.

13. Bhattacharya D, Price DC, Chan CX, Qiu H, Rose N, Ball S et al. **Genome of the red alga *Porphyridium purpureum***. *Nat Commun* 2013, **4**:1941.

14. Nakamura Y, Sasaki N, Kobayashi M, Ojima N, Yasuike M, Shigenobu Y et al. **The first symbiont-free genome sequence of marine red alga, susabi-nori (*Pyropia yezoensis*)**. *PLoS ONE* 2013, **8**:e57122.

15. Price DC, Chan CX, Yoon HS, Yang EC, Qiu H, Weber APM et al. ***Cyanophora paradoxa* genome elucidates origin of photosynthesis in algae and plants**. *Science* 2012, **335**:843-847.

16. Curtis BA, Tanifuji G, Burki F, Gruber A, Irimia M, Maruyama S et al. **Algal genomes reveal evolutionary mosaicism and the fate of nucleomorphs**. *Nature* 2012, **492**:59-65.

17. Gobler CJ, Berry DL, Dyhrman ST, Wilhelm SW, Salamov A, Lobanov AV et al. **Niche of harmful alga *Aureococcus anophagefferens* revealed through ecogenomics**. *Proc Natl Acad Sci U S A* 2011, **108**:4352-4357.

18. Cock JM, Sterck L, Rouzé P, Scornet D, Allen AE, Amoutzias G et al. **The *Ectocarpus* genome and the independent evolution of multicellularity in brown algae**. *Nature* 2010, **465**:617-621.

19. Corteggiani Carpinelli E, Telatin A, Vitulo N, Forcato C, D'Angelo M, Schiavon R et al. **Chromosome scale genome assembly and transcriptome profiling of *Nannochloropsis gaditana* in nitrogen depletion**. *Mol Plant* 2014, **7**:323-335.

20. Radakovits R, Jinkerson RE, Fuerstenberg SI, Tae H, Settlage RE, Boore JL et al. **Draft genome sequence and genetic transformation of the oleaginous alga *Nannochloropis gaditana***. *Nat Commun* 2012, **3**:686.

21. Pan K, Qin J, Li S, Dai W, Zhu B, Jin Y et al. **Nuclear monoploidy and asexual propagation of *Nannochloropsis oceanica* (Eustigmatophyceae) as revealed by its genome sequence**. *J Phycol* 2011, **47**:1425-1432.

22. Vieler A, Wu G, Tsai C-H, Bullard B, Cornish AJ, Harvey C et al. **Genome, functional gene annotation, and nuclear transformation of the heterokont oleaginous alga *Nannochloropsis oceanica* CCMP1779**. *PLoS Genet* 2012, **8**:e1003064.

23. Bowler C, Allen AE, Badger JH, Grimwood J, Jabbari K, Kuo A et al. **The *Phaeodactylum* genome reveals the evolutionary history of diatom genomes**. *Nature* 2008, **456**:239-244.

24. Lommer M, Specht M, Roy A-S, Kraemer L, Andreson R, Gutowska MA et al. **Genome and low-iron response of an oceanic diatom adapted to chronic iron limitation**. *Genome Biol* 2012, **13**:R66.

25. Armbrust EV, Berges JA, Bowler C, Green BR, Martinez D, Putnam NH et al. **The genome of the diatom *Thalassiosira pseudonana*: Ecology, evolution, and metabolism**. *Science* 2004, **306**:79-86.

26. Shoguchi E, Shinzato C, Kawashima T, Gyoja F, Mungpakdee S, Koyanagi R et al. **Draft assembly of the *Symbiodinium minutum* nuclear genome reveals dinoflagellate gene structure**. *Curr Biol* 2013, **23**:1399-1408.

27. Read BA, Kegel J, Klute MJ, Kuo A, Lefebvre SC, Maumus F et al. **Pan genome of the phytoplankton *Emiliania* underpins its global distribution**. *Nature* 2013, **499**:209-213.

28. Hori K, Maruyama F, Fujisawa T, Togashi T, Yamamoto N, Seo M et al. ***Klebsormidium flaccidum* genome reveals primary factors for plant terrestrial adaptation**. *Nat Commun* 2014, **5**:3978.
