# Supplementary material for: Multimodular type I polyketide synthases in algae evolve by module duplications and displacement of AT domains in trans
Source: BMC Genomics. 2015 Nov 26;16:1015. doi: 10.1186/s12864-015-2222-9 (PMC4661987; doi:10.1186/s12864-015-2222-9)
Supplement: Additional file 3: — Amino acid sequences of newly annotated type I PKSs. (DOCX 25 kb) [file 12864_2015_2222_MOESM3_ESM.docx]

**Additional file 3**

**Multimodular type I polyketide synthases in algae evolve**

**by module duplications and displacement of AT domains**

***in trans***

Ekaterina Shelest, Natalie Heimerl, Maximilian Fichtner, Severin Sasso

**Amino acid sequences of newly annotated type I PKSs:**

1. CvaPKS2 from *Chlorella variabilis*:

>Cva_PKS2 ChlNC64A_1:g21.t1

MAVRLAHGLQNRRVIAARGVLPLLNYTSLRTSGSKHVTSARFDVLLRCGERPEVWSASAWTTTPSPASWMISTMGSGSRCELLQGKEPAGCGKGGGAWPLDFHPWNHCKLWFTAGVLNPLPNVCVLNPLQEPPIRSMEPPRLPRAVLLAGVVWRGPPCNACSDACRVVSAPLARDAVAFNQKGQLGLCKNDVFLGGCRCRPTGGMWISGGWERAPCPSDLVAFWREPNCSTTSSLLFPRKRHAAAAESCCNHRITRSMCPSRSHISTPSPGFSHSILHMLATAVLQATAIDAQQRLLLEGCWEAMAHSTPGSGAGEACNDARAVGVIVGISYNEYYLNSAHQGMSAYTATSGTLSVVCGRISFTLGLKGPSVSIDTACSSSLVGAHLACTSFIPGGCYRSLAAGVNLTMRAETTAVLSKAGMLASDGRCKTLDAAADGYMRGEACIVHLLEALAASDGVAGSVLLPAAIIVGTAVNQDGRSSSLTAPNGPSQQAVVREALACNSVAASDVSVLEMHGTGTALGDPIEVGAAFAVFQTVGNHPLELQAAKSRVLHTEPAAGAVGVAALSLHLGQMGRHDILHLRTVNPHVSSVLQSYAHQSDTRGWLARRQGAPVLHSTPEMHGSVSSFAYQGTNSHAVLGMTCPMSCTAPQTWSWQHRRLWYQISSHPLLSQFIRGQSGTATATALANIRMQCSLQRPALSYLLQHSVCGQALVAPTVLLEMAAAAGQLLWSQEEINNPVALTALVIKQPALLLDGTVNPTLNCDISPASGGIIVGSGILPSGEAAAHIHGRLQGIDTAVTRLEVDVLSVDAWLLESQGANRATNLPPHGPTDLNGMASLFNPTDRKRTWALFGSVCVKEHRNSSCWIHPAVADSSLQLNATVASQSQIRSVGGAVLAACGAYAPMRRLAASSAAVGAAVMQDGGSSNTWLLSLRQQLLATLGNQFKALKAAETPSPSSGAVSPCGLPAISNVCRPPPIANLDGITQQLSEIVAHILGTDGVPADQPLMDAGLDSTGIVELTNAVQDVFRIDVPASITFDYPTVLALADYIASEGLASTRHQKVLCVVSSCNKMPSSEQTRQLTSEVIGWASSMASNGNEETSIVRRMLTSVDCVAPVPLERWDIEDMGGGKDKLQASKVRFGAWIDCISKFDEGVFRLSRAEAIGLDPQCRMLLDNTFAAFQDITPPVPTTIPSSSRGRVGTYVGCVWDEYQELQNEHCIQLSIAALTGSGLSFLVGRLAYVFDFQGPCIGLNTACSSSLVAVHLGHHALQDGEATSSATLWAHSGAPIACVSSLHGLLALFQQHAVESIYGAPIACFSSLHGYLHFLATRLTAGTNAILLESTSRHLAQLQAVSSDGRCKTFDATADGYGRGEACIVFVACSPDNNQHPLAVMHGSAYNQDGRSSGLTAPSGPAQTALVRIALKKSNALPHLVGWVTTHGTGTPLGDPIEVNALGQALAASAQHVTLCSLKASFGHTEGTAGVHGMLITTFGLQYSSTPPNIHTRNLNPYISSAFQQWHGYNTVPFVARTPASWHAATLSMAGCSSFGMTGVNAHALFSAIPFQFHCSYLLIWHRQREWPVPSNHPLLKSLVWKHEVGLARFQCWLSQPIMADIYDHTIQKTSILPGASMLECCIASSYILLSETIAMHTVCLVDCSIPSPVLIHTTMPGLLDCDIDVSGIVRLHTSNAQTNMAAKLHFHAIVHKLADYCCTQSISPNNAVVCSSCKAPNFMPAMENISGRLRSLLASINAEYSRFGTSNVDLDFQWRGNGWIVHPAIIDNALQLGPATASIEKMNKSTWVMPQWGAVSSSMIREQTRTVDVIRTSHWCSSGNGHFIHVDELQAKTIHLAMDYQNIISTSLDCHRMIYETEWQALEIHVLPEEHSLFIDPWRLQPKISIRPTFGPSTEFVVTCPNWPAAETCESAAAMLTCASMTMLLQTAEAGPGGAVQLVSQAAQSAGRTPSGRGCSVPVMGMSSMMRVAAMEFGEVHWAWTDRDDAYPALTPSGDVQSLWQMPDGQSDMYGRFLQANTWVAPRLLGRDPGQTDKGGFLSSSMAASLLSGTFVVTGGFGALGTFVALWLNGLGVSDVQLLGRSGRTSGRSMAAYLLGSNCRLVCSKGDVACSEETDYVLTSANRSKDKVLHGIMHAGAVLDSKIIANIAIRSIRNEYSGKVYGTHHLLRRSFTSALSVCKLFSSLAAFGGAAGQASYAAANGSLNAWAKNAQGQGHVGLAVQWGNWDGGGMAVNNKGFIERMKRIGLGMIDPDVGLSTMACLLHQAASNTNKWSWHQEVFIGTVFLWDIILQKFVHIPMYFQEFEKGSPLDQQNYSVQTPPYLRMTTGALHTTNVVKVLHPVPQYLEQKQHHMLTHILNIMEGLSIRNVSADQPFMLDSIGVVQLRNQVATYSGVEVPPTAIFDHPTPIELARFVVGQVSMSETQMPNFAKIAKGQKQTLGIQTVSIVNSRVLRDGIVAQMSALVRMIVGHDVGVDEPLMAAGLDSLGSVHLRASILEQFSVDLPATAALDFPTIAALAGRVAACNPTHADMSTVQHNSLDPSNISGNNNPVTGIVGLSCAYPGPAAADCCCDFWEAAVLGENIQTTIPYNRWDVELHYSPEINVNKMNVRFAGFISNLEFFDSEMFRFGRNEAKVMDPQSRLVLEHMHHSFIDAGTRTGQSIAQDAGVYVGVMHIEFLSYLSYMGIAISPNVVTGNGMDFLVGRLSYTFGLSGPCLSTNTACSSSLVATHLANAALVHCESHAAASCGVFMVLLPGTMAGISQLKALSPEGRCKTLDSSADGYGRGEGCTVATIELVDHGNLAIAFICNTVVNQDGRCSSLTAPHGPSQAALITRALSNAGMPPSILKYLAIHGTGTPLGDPIELSALGQALSNASGLQYLTIGSVKSCYGHTEGAAGLTGVLLLLYAMGNQCTTPTMNLRHLNPYVDSCINDWQKMCHLYSHASREAAPCDIPTPQKLAGTSSFGMSGVNAHALIKGSGTVGQECRYAKPSTIRKEWLWCLTPAYYILGKNTPARTVQKSTFMLSTCKPELAYLKDYKIEGQALLPSSAMAEASAAACLTLLDIQQRTQLTLCNTCVNPVLLVKHSAVLEVNINYTNGDMQMQTVTTANTITLMNYTCGLITPAWPDFEQELSHITATDRMKHTTNLSALLNTLLLNKWVVVSDIFTSNVDQHPGYCLEPTSLDAICQLACALTRRSASLKLPNFESILIVQSKSYNNIKQMSAAQLIAAKSGDETHIAVKCSHTHHTPPQQCLFALRKIQLQSTGNGDGNSDIDRQQQDWLYCLEWTVYGNVTAALVFKAAEVNCVQLAMADGHASTVATAIAVGQQAYVGGAHCIALLTRGALSISGAACVVPNANLQAIGIWAFARTIAQESRSIDVQALDLQQQYAGSHEMKTALLAATSITDRLLPAQVHGSAFGHTLQGGTMAASVLQRVSVNRNTPATLLFNNATSLTHRQVVVAVKAIFQYSRPVHPLHFSDSPGESSGLVHWAGIIVASDPDEDHLRPGDSVFGLNSCCIGAHIQTCAQNVFHMPINTCFRDAASTSMLYVTLAVALRDMASVQPGDNVLVHMMDGRTTALTAQIAASLGAAVLTTGGNICDRAWLRGLGVKHVMSSCCEAIISNTLQLDTPSVVIKSFAESDTNAGCLAALRADGRFVVINDYNMWSKERVAQERPDVSYRASFMERLHVSMDLQPLVYQMLADLTFILRGFTFLTNCNIPILGTTFDAGVEKGYVVVTGGLGMMGSLVGIWLAMEQMPQIVLLSRNARPRIETATVLQLGSGAAAAFHIIQCDTSNAEEVSVVMASNQGRRRLLGVVHCGGVLKDATIPNQSLLSIRSSFAPKISSAYHWQLPVSLKASTLHLTCSSVAALLGSPGQANYSAANAVLDGMALDRQAQGLAGMSIQWGAWAEGGMAASNAAIAIAVQRLGMGMISPAQGMAAFQLFMLMHSATSFPGVIVAVPFRWEKFIRQLDTSAIPFVLHGVATPTCSEHEHETAMLGHTCGHPDISNGRAPSASRQTSGTPFFNHTESSTTKEKTKHITEQLFSLVTIMIGTTIDLQQPLMEAGLDSLGVMELCNNINLVFNVNVPPTVLIDYPTISALSGYLAQATSDCPDTYSVSLASGFDDSSAHLADFHVELASELFSSDEFLAHNSLAVIPMERWDVDGTPSPVLDYNEIRFGSFVPDVQLFDAAAFNISSVEAVYVDPQQRLLLKDAAEVLQEWDSRPHQVSTNMPIARERLGVIVGVGPADYVNLTTATLPISPYLITGGTISVAAGRMSYTFGLHGPSMSVDSACSSSLVAVHYGVLDLQAHHPDATLAAGVNLLLLSHRSTALQMNGMLAMDGRCKTLDASANGYVRGECSIVFLLAIVSPSMLDTPQSLPSLLILGSCVNQDGRSSSLTAPNGPAQQNVIKAALVAGNTAGGQVGVLEMHGTGTALGDPIEVGAATRIFQDEHRSYPLSYTAVKARVGHGEIGAGILGMLQMWQQLAHCMAHSITNLQHINTYISRIFHEEIKSGVMTQVPRQEVPLTLPSNKQCASISSFAFQGTNAHVVLSKSFDLSRTRQPNAEDPRMWNRKRFWTGPAHHCLLCCCIPTAESNQRTITFQAYLGRAPGFLRQFDNGITPASTLLEIAAASGKLICGDAQLPSLVVIELAVAAHMQGKEQSQLLSCAIEKQFATAELCQTNISNNRITKLLTYMLRTVTVQQLKKVSHKKPAILFDFVKPTNMELPVPILTAQVARMACSAVNSPLLPFSFEEAVIMAADDVESCTKGVSACSILKPYIRTGTSLTVASPFQTIIIKSNTSHRVELQTITSSAYDSRVMDISGLQLTPKRGRQAINEYTLFWQRMESTQCSGSKDICDSTWLLVGIDDVALSSICNVTAADDVHSTSFTYWYTPMWMIHRPTVASELVGSSKVHLHALMSIVTADHILCVQPEHWHNRVDHATLCGVAPAFTVSKQTSSMSENEQHWQQLLYVISLAWAYTSKGMAHQNCKASFISRGLRHLPGWFSCNTNFSSASGAALGSLACGIGKSLFMENRKAYGVALDLDNSGTHLSTAQLHWLLQHSEEYFCAVRGGHLFAERLVKHWSLPRQRQLAIQACMTCIVTGGTKGLGWMYARQLIQNACQTLVLTSRSGLLSLADFVSLSSQGSTTFVAHRDASEPVHGRQLAHWLHNHLPAVQLCAHAAGLPGFNLIDDLTCEQFIQVVSPKVMGASALGGSHLTVLSTVFFSSTSAVWSQAGAAHYSGSNCYLDYTAHAWQGAGQPATSINFGPFGDIGMAASLSESMAAVGFRLQDGAQLFPIFARSAYLPQSINARLSLGKFSKINTTKGRWALIDDLTTIPESSATPPQLSQVPVAAVSTHPLFAAAAGPASMATAGRPLLTLGEAQHIVHTIAVEVLGEGQLGSSGQLPAGGLDSLSAVELSNQIGQAVGLDLPSTLVFDYPSVGAMAAYLMTKIWPKHSAVASADTAELTQQTAVHSAELLLAGAPPDPTTEHFAILVSVSARLPHVQLQGSPTSAPALDAVSVVPFDRWDLEASWGSKRGLRSRFGGWLQGVDYFDASLFTLSGSEVDVMDPQQRLLLELSWELVPRPDSGRPAVNACGIYIGIQQMEYGSLMASHNGTLGAFSGVSNSCWHSIEATGTPFSVAAGRLSFIFGFSGPAVSIDTACSSAMVGTHMAAQHLQQHMGDALSAGVNLMLAERTTLAAQAADHGGRHRHALPFCPGMLTLDGHCKTLDKAADGYVRSEACVVLHLSASNSKDAENGASSALIHEGVVLCGTFVNQDGRSSSLTAPNGPAQQRVLRGALQAAILLPEHVTGLEMHGTGTALGDPIEIGAITAVLPGAGLPLQLTAAKSRLGHAEPAAGSIGMVHAAVELCTVHSSSITCLTTVNPYVSSTFTELTTIGQPIPHISRQDAAAVALPVMSSMRATMGISSFAFQGSIPLMSKLCEDKRGIADRLSANPSQFLRERLIESPRLRHRPPRDPFTTSGGGLFPTVIGSKAGASWRRQRFWYAVPQHHLLEQCRFNAKTWMASLKFSSSKASLAYLHDHKVQNQVLLPATAMLEMVLAATSSASQVDATLCRLVFSASISSPVIMAIARDVALSCSLHPTTGSLEPFILMLQIYIVDVYCSTNTYAGILLESLGARKADAPSLQYRMFAAFVASASDAFATAIPHLLHPTDTDTMSLSSRRRALAAGQVDSSDLLRQQPGGYLMHPAILDATTHTAATLQGTQKTEAGITRVPVGVHALLARSVPLGRTARHWCQGTILASAGNGVALTNFAMAVSGDSGIYISGFEAKRISRHAFGSFEKPTTSRSSIAQVGSEDAHARETSHMLATRANVSTLQQLVHQIAETVLGAAMPPDQPLMEAGLDSLGNGISKEHFTLSVAGAMELRTSLDAAFGVDLPATAVFDYPTVRALTQLIAQLKQESSGYPIATESGTASDQHGFNTTVATSGPAFHSVVNAINRLVQRVLGAGDAVDSEQPLMEAGLDSLSAVELRTALNATFDLDLPATTTFDYPSVTALAGYITLQLKDAAEAPAAHATAHAATAGQATLAFHADYAGVPKHLSRSVAAFQPTMRYTAVTGTAGRYPCTSEFGGLAPFWQLLEHGANLPRLMPLQRWDVDQYYATQDEGNTRTMYVRLGGFVDSVDVFDLTLFRLAATEALAVEPQSRILLEQVFLALADAQPATGPLLDSRTGVYVGCMYSEYTQLQHSLGYKMSPGIVTGNGISYLVGRVSYSFGLQACRLPSRVALHAGPSISTDTACSSSLVSAHQAHMGLLGHETVAAVAAGVNTMLLPITTTSICGLGALSPSSRCKTFDTSADGYGRGEGFGVVVLAHASSNANSRLQQHDALGLVCGSAINQDGRSSGLTAPNGPSQTALILVALAAGSLSSSLVSYMATHGTGTPLGDPIEVNALGQALHPAHSSLRAPLTLGSVKSCYGHTEGAAGVTGIFLAAKVLQQHSHPAVLTLRSVNPYVEAALRDWRKRNVAVASVPKEGSAGSIWRPELAAGTSSFGMSGTNAHILLSVPNARHVPNLINIWRQMRLWPATMPSHLLPRAAVHLRQRQCRFEAYLMAPALSFLWDHQVQSRPLLPGSALLEMAMAAGHMLATSIMQRDSETSTLLAECTIPASATLSDSPAAMQLQLVLGLREGSYTISSVHSRITHLAGGTRNCSHDHRSKTFASVVPANTETTVQLSAMLAAGPVREVRLASQLGCLSINPVVHTDGYCSHPAVVDGCLHLGASLAQSSEGDMSRITVPVVVRSYASTREFKPNADMNTYASCRLSQISLDGASVSSYSLKSAFAKTAVTEIADLEARPMRVMAATRKLPIFVRKESSAVVSNEHSGEVGAIAPRLLYHVLWEADSMTGQYNQEARQQQEHANAIALAVKVHINTLMQLVATPARSHSSSADAAMPTSQLLSLLQEVTGRGRRGTQIRVATTGAVRAGPSLMGPSAGHSSAAAWGMVRVAASEVSNARWALVDLDSQASISQTAAGEAVDVSGSLVRHGVTFQPVLLPLTSRQTATPTLETAQQTMMLITGGLGERPAVERYYTGNHAPRKCDASAGIGSLLSTWLAYQLGAHLTLLGRTGYFTSSTPDLMLSEAPVTALRCNASLTAEASAAFFSSTTACRQLAAIMHAGGLLQDGMLQAQSAASVRNVFSPKLRFVAHAANAARTHAMHVVNLFSSVSAFLGTPGQSNYAAANSALNAWAGVFQQCGIGGKLSIQWGAWATVGMAHSNAMVLMRVDKAGLGVVMPMHGLTVLQSVLEGSAVSQLAEAIATPFLFDRVLRGLESVPRVFLHVAASISIWPETSVDSPVIDAREHEMAAATVSLKSPSALGDIILDVRARVHAILGSEVSDVQPLMEAGLDSLAVVELRSEISNTFGLELPVTTLFNYPTITALANFIAAQLESLSGMAVDADLATLELKVVDAIAVEAEILRIVRGMVEMEISSSQPLMEAGLDSLAAVEFLHELEKRFEIDLPATVMFDYPTVAALAQHTAGILAAEGGTLASGGLATAGNLAAPSLPAQQDTVTNLVGLSCRYPTTSNSLVSFWQGALQPVDLPETVPLDRWDLDRLYAPQPTNSTMYARFGTFVHDVDKFDWQMFNLTRTETLAIDPQQRLLLEESHACLMDGRPATGSLFGTETGVYVGCMYQEYTDVLSSAGTKLSAAAATGNSRSFMVGRISYIFGLAGVLNPGAAANASPCLSTDTACSSSLVATHLAHYSLLNQETVAATAAGVNAMLSPTTTVGICQLQALSSTGRCKSFDSTGDGYGRAEGFGVVLLAPPHPGQLAVAAVRGSAMNQDGQSSGLTAPNGPSQTKLLLTSLRRGGLNSMKLRFIAVHGTGTPLGDPIEVGAIGQALQSGRNKTAMLTLGSIKSCYGHTEGAAGITGLLLAAQMASRLCVAPIMHLRNMNPYVEAALGDWQNHSHIAATVPRQKQGGDEAALLAGTSSFGMSGVNAHVTVGSVGVASQHAHAGNIVLKQTFSSRRIWPLPLANPILLSILPKQHQAIFNCTLSHSAVQCNWEHQVEAVAFLPTAATFDLMAAAASCLTEASNAGQALADVILMVPIYCTLESAVTCSILFTSGSFQVAAGTEVSATASMCQVVSPSSSTGIVKAPTGITVAKSMTACATEGPTSCIFTAVVGQLEHCANSHNRCYVYTQAAISLPGLPVPQTVLGCRLYLSAPAPASSLHPRAAIASGQNILLVTNNCPAACQLHGLLLRPLPAVSAKRIATQRQSPAWQLLWRPTEILPVQQRFRPCLILSTHPCYLSSLCEYSSHLQGGDVLLAALNAVWNENSGTANNASSSPTCCSELCLSSEVHLELLLHTLGSQQLCFLVQESGSAVDLSAALVSYRAVARLSTGLRIALITYNQQVVGSLGPSTSSQVAFLQGEYQDIPCKSKRQEEEGLARTLFMENRSAFVTSIDLQKFVPPPPVTTLTSITDQAYGFEVAFRSGRVYALQLTQGATPRARPYAKISTALVSGGSKMHCWSGASVQVTVKGVVCVFEVGLGHEYCQHLVHRGCRMLVLSSRGGSLSLDAFCNLVASGCSVFAIKADSRCRHSMGQVLGWVREALPLIDHFAHAAGVPGFAMLRDMSTTEFREIAEVKTAMTSAFQEAALPLQSQLAFSSTSSVWSQTGAAHYASANVFLDIYAARTQQAGLPCTAIQYGPFATSGMAATHVGGLEALGLKGLRPQQITESRLVAGSVSHMLYARIDVLRFSTIYSAKGRWGLLDGLLLTSQGRAPSPSANDMPVCTVAATNGSFGSSKETNIAPVGLQQVEQAVKDAASDILGVDLDGELAAGGLDPSGQTATGTGNFPAGGFDSLSAVELSSTLSSTLGVQLPGTLVFDYPSVQAMASFIHGIMGTHGHAPDLAASYASMRAQQLPISAGFGPIDAPHALHSSRIASVTMASRLPMDHTVCGRDADGICSVPFERWDLEALRIGKSVLRTRYGGFLHGIDQFDASLFGITSAEAELMDPQQRLLLEVTWEVVRGLDTRSRNSAPESAVYVGIQQMEYGNLARPYLHTIGPYSDPVHPHTHALTSWSCHPSILRAATGGSFSVAAGRLSFVYGFKGPAVSIDTACSSALVATHHGMAQLRSLPQWAVLAAGINLMLSEATTAAAQSAGMLTQDGRCKTLDAGADGYARAEACTALMLADGADLSNHIEAAYLQATFINQDGRSSSLTAPNGPSQQAVIRGTLQAASLQPRDIVALEMHGTGTALGDPIELSAALAVLHYGSTPLQLTTAKSRIGHSEPVAGTVGVTHAATMIAYNSISGVMHLQVFNPMLSTLLHGYMAGSRVAHASRQGSPATVGNTLSSPWQAACGISSFAFQGTNAHAIIVKGPGQPATQQCRPIEWQRRRFWFSTPPHALSRALTGARHVLQVQGVLQSASTAYLWDHHVQGRRLLPGAAMFEAAHAAGALLLISAANDSRKAGTAGSATPALVSVSIPAPLRLPACSGSLPNFVILAVAVEAATGRLKLTSSSTLSEASTHLAGSYACSVEEKDATSQADLAVHGQTTFSLLFSCAEWATAGGSAQLPSGIAGIQRSKFFSQSGQYHVHPAVTDNATQVRTNTLLDLAFLHALRMADCFFMNVGLSSLPLCCFLLQIGSAFKSTARSAIIRVPAGLSVFHVATAEHISPRATWAASAALMGSTIPDGAVACSYRVATENGEPKPQLHIGAMLFKPTGNTQSAEVAPSSRKTADGAHTFQSAKLMYAIQWEASIPIDAGNPAVCSIVQHGLLWEIGNSSGLQLPVRGASNAFEAVQNSLKLLQTAMRGRSEAAVIVNSQLLTAGTRKSSPAYAAVSGLLKVAAREITDLHFAQFTHEAFAAATAKLPAESTDAFGTATAKLQHMETARQTAPGSILVSGGLGDIGQLAGCWIATSHPHVHVWLLGRSGRFTHPMAAMLSSHASCITMSASDIAVTADMAGLMQGLAASGSQMVTGILHAGAVLHDTLLSRQTAQSLRIVFAPKASGALCLLRVASGMPLDRVVQFSSLTAQLGTPGQSNYAAGNGALEGIAQDHVNSGIQNSSIMWGPWATGMAGNNPRILERFRKSGLGVITGVTGMAILQAVTCSLAQMPAVIGASILWTQLFRGAQQVPSIFADFVPANTSGSRNIAALLDKARPVGDDNQAVASPGHATATHIDVLGLVTTMVNGMLGAAVPPDQPLMEAGLDSLGAVELRNSISDALGTELPATLTFDYPTISALAAYLNLNHAPSDNNAHEPASLPNTVLDAGAILAELHSIVKGMLGIDVTSEQPLMEAGIDSLGAVELSNTIAKRYKVDLPATLVFDHPSLEAITSHLTSFLKMSDFVDALQESSLSDVLFSRLLPPAEAYSTTITGMSATFPADITGMSAFWAAANDGANLQKEVPLGRWEIDAGYHPSTTPQAMTIYVRFGAFCSGVDLFDHGLFKLSRNEAAAMDPQQRLLLEGTLVSRTEAVSSTYIIAPAASIPSPPAGVYAGCMYNEYTSVILSGGHKLPPQAVIGTGLSYMVGRISYTFGFTGPCVSTDTACSSSLVACHLAHKGLVLAECNAAVAAGSNVMLLANTTAAICQLQALSPVGRCKTFEASADGYGRAEGVATLMLCRQQLAQLQHPRTSETPSVIVQGSSVNQGGRSSGLTAPSGPAQTALVRTALASGSLSTSQLCMLSVHGTGTPLGDPIEVGALSLAMSGTSSGGIAAVTLLSNKSCFGHTEGTAGITGLLLSVAALHKQAAPEVMHLCNLNP

2. MccPKS1 from *Micromonas* sp. CCMP1545:

>MicpuC3|scaffold_15:58957-81298:g1.t1

MAASAEVERRRRTLASVSAFAFQGTNAHALLEVMRGARVIGGGAATAFLDRGKHWSMPSPHAWVDAAAVILSDVVCVAAVFDVILGGCNARTSTVTLWDHVVHGKPLCPAAAFLETAVAAADRSYTNRPTASDAADARGGTPGVRFVAVPSPLVLPARDDAAATTTMRVTVRDDRVRVESSARSRSFENDADARLRRRVSLHADGGLLCIAPRRRPSRADAADALVVLFSPSTPAPSSRPRAFASLTCAHASQAGSFGVDPAMLDAGIHLAEGSVSTGTRAARIPSAVGAFCIRDDDDSRHHSRRRFASCVAREGDATAREAINDHVVGGRATLGGLRLTRPRAVDPATPGLEPSRRFAVETVETVLYETRWRATPSCLAAAGPETVSKRIVSATRPRPRRAAVTAERTASTALAAFLASSRAPSSSALLRTIGAHRPTANVAPSSASDASDSAGVWGAARAYGLEVMGTVDAVDAAPPAAAKAKAAAKAWTARRRESDRIADALTRTTRGAGAAHVARLLPRVPASAGEASSSSAVTLLERVIGRAVLRDGGGGGRGGFSAVAAAFRRPPGPAVGGAAFAAGGLGALGVVVTRWFLREGGDVAVMTGRSGRQASTPLPGSKPGALATTRALFVAVRSDASASEEARAALDILHEIGGGYTNARGLTVFNISGVLQDAAVANQTSGKVRVVCAPKTVAARILFDRALQVPISSTVAFSSVTALVGNAGQFGYAAANAMLDAKALAHANAGVASKSVQFGAWAESGMAADSNLSERARRAGLGLLTRRDGLRAIAEALRVDDVGVSDDDWNTSAASTTIAACPFDWERYKARHARASASSFLSEVGGDAAPPPPVRSAVSDAASVNAQKTSTVTGGTPTTDDPRVTARAIVHDAIRAVLGKPLDDDDAPLVDAGIDSLSTAELAAALGDGIRGLPGGGGDALPSTLVFDYPTPRALVAFIVAALPVTRAVVAIASNEDVGRESSNGGAASSNRRFLRGSGGASVVVAVGGETAAGDVSERPSASAYASASAPPSDAVRVLPLSRWDLEMDDWNGGGEAASGSVAPRFGGFVSRVARFDADAFGVNPSEAHAMDPQQRRLLTWTSRALSDAKLETPQRSTEEPLVGVYVGAATTDYGKLASDADATPSATAATGSAFLSVTAGRCAFTLNTRGPAIAIDTACSSGLVATHAARAAIDRREDDRGDARRRQLQVVAGVNFLLHRSTTAMFHAAGMLAPDGRCKSLDASADGYVRGEACVVLALDCGGGCESSHPGGGNESSHPAAAAAAVVVVVVVVGSGVNQDGRSGSLTAPNGPSQRAVMRAARREADAAESATGTAPRRRRLATLQTHGTGTPLGDPIEIGAAVAVAADDDDPDRVVAAADDLLLGATKASVAHAESPAGLIGLLAAITTSLIGASNDVNPHLRWVNPHVAGVLEAAKKRRERSAAATRQRAASASFLGRDALAGASAFAFQGTNAHALVAKNNLEGITDVDGALRMWSRVATTRAERRFWLAPAPHPLIVRASADGGGGFARFHVGSIANAPRASSLRDHAVNGRSLMPCAGFLELIRAAVAFSISSVESTAAAAAATRVVIASPLDLRAPDDAHVRVRVDRTTGAVECDAGTGTGTGTRVKASTTAVAAFPTTATTTTATTTTAAAGPGPPARTVFGRRASFLEEATTLLASLARASSDDNDDDYTSAVSPAELDNAFQSVSAAWTPRGGTSPPRGLNVPTAVDAFLATAASSDCLPVQHASSSSFASSAVIDHGSRGYTSQHGVATNGVVVRGVRAKPMRRRAARSFDSDETAVVAANAIDAPELTVLIPASQATPSRHRGRSNREEIANSPAAASRSPFGAIARSCACVYAAAAAAAAATSRDGESLSGVTVAGGERLGVDGGARAMDAELGSRVRVRASSLDGRARVAVRAPASDWFSVRRTTTHRTLARRLLAPASDCLPVHVRTHQKSSAPPSTFDAAASSSSSSCVVTGGTGALGVVFAAAATDAWGCSRVTLLSRVGRRDVAVAATRGAGVTRVVAADASTRADTSAWHDAIGWRSAFCDPPLGVLLHAGGVLRDAAATNASAGAARAVVASKKLHDGGRFFLTQNPARALVFFSSIASCVGSAGQIPYSAANASLDSASATMTREGLPAASTQWGPFDAGGGGMVDAAAVRASLARSGFGLLARHEGVHACERVMCGLENGRRDGVTFAAVTCASRTDWAVVASRRPGADVARFLEDILDDDDVGANTRAIDQRRTLLVTTTDAPRVDMNRHDVNTNAAAVRIPLRDVLDAVRSVVASLAGRDVSADEPLMDAGLDSLTAVDARAEVSKTFNVTLPATALFDYPTAEALATRVWGVLAPPPAPAAPARAVVPENAREAAAAAAAAVVAFSSAGGAGPHDAERVRTIPPHRWDVETARVRGVLPAAFGSFLASPETFDNDACALSLTEASVVDVQQRLALDAISTFALLLVSDDDETGGKRAFHGTDTGVYVGVASRDYDDISKQVGVHGQWSPSALAAAASFGSVVPGRASFTFALTGPSIAIDTACSSSLVAAKIAFDDVAVAALPVCGAVVLGVNVTLTPRVTETFRAAGMLSPTGRCKTLDASADGYVRAEECRAVSLKGTSASASANASFDGPGDAVVVVGGAGVNQDGRSSSLTAPNGPSQQAVIRAAMTDDDYVLIHHHARVLHMHGTGTPLGDPIEIGAAVAVFAQRGVMGGETRREKSLRIGVHRADAVVREPVTASSPPPPLRLESSKAWTGHAEPAAGALGLVVLCANASEGKTAGFGGTLRVMNPHVVACVRGGGGGGGIVVAAPRATAPLPMRSDASCDGVVRGGVSAFAFQGTNAHVRVVVGTTRRRNDRDRAAGFWRDEAISTTAHRRRAWIAPPTPLSLSSVAMSDEVNDAARFDASALTREPRGNAFEDHVVAGEALFPGAGMLHASQEAAAAASGSTVPLLLLLLRAVIPAPLLLRGCNVRRLHVTVRLASGLVTFRSSREGRETTHASSTAARAAISARGGGGNYRPTAISLDARRASHAVPIDAAAAYASIREVGLQYGPRFRPLHAIRGEETSVGGGGGGGGGGASRVSKFRVPRSCGVVASTAVVVDGAMQLSAPILPTSSYNSRRLRVPASVGAYASPGGGGRNGDGDAGWVYFCGGGGDDGDGDGDGAATDHGCEWDGGGGGGGGRSSATARLVELVSRPLPSIASSVTPAGMTSRSRGRLGGAATRGVRYELHARAALVVRDADADAERETVRVRSLAASRRRGPVLAMLPAGNASVRAVAAAVAASHAPARWVATAGGPSDGSGLKFSARGAAIGACVRGVALSVALESPPHLFAENEDDENDDDDDAIRETTDRRREKRVVAANANVLLPSFPRVGRRPPTPAPQVRSIHWSPYDRVRVRAFLAALFGPRRNASVASVMLLLDTVQSHGGSYDAPPPPTSTTDAFRIVGGLGAVGTAAAERFAAAAKNTTTTVRMTLAGRVGRVASRAVPGSGPGSSGTDTAFPTATLVTAIKCDGATAEDVDASIALAPPGLVTIHAGGVLRDGLAASQAAGAARGVFAPKTVATGAFLASTLCGTARAGAGDILLSSVASLLGSAGQSNYSAANAALDAAAAMARRAGAAATATAFGPIGGGGGGGGGGGGMAAADARTTARLERVGLSLLETAVGVGAIEDVVDGGWAAASAASVVVAAFDWPVATRAASEEVRAGVLREFASDDDDSEEDCSIVDAVVVTTKRSNVSRYADVAGLEAMIAAETKKVIGDGDGEGAALDRDAPLMASGLDSLGAVELRGAIAAKVLGGVDLPATLLFDYPTIAAVATHLHRRFGGGDGDGGDEASVAPVRVVGSDGGAAPRAVAVADACTGGERASSSRDAVAPIPRDRWDVDARGAAAGDDAPPPPGSRFGGFISTSSLTAFDHDLLGVRVHAEAMHLDPRQRVLVADVSAAWTAAATTTAKQRTNDVAVFTGVAGKDYALLLKSCGVQIGTFTGTGNEASVTCGRVMFSLGFTGASAAIDTACSSSLVAASFALDAVRRSFDGLGRVDRAPRTAHAFAAGASLMLTPDMHLTLGGAGMLSHFGRCMTLDASADGYGRGEACACVLIACGDANEREEDEDTIKGDAVVAFAGSAVNQDGRSSSLTAPNGPSQRAVVREAAFGFGSGSGSGSGAVTEERVGGTRLLQMHGTGTPLGDPIEVNAAVDAFFFLHLRLRRRTSSSPTDDASTATAVVLVLEAVKSWRGHAEPAAGVLGLATLRSQLAELATHGQCHLRAMNPHASDASLKAFGRGGGVVAPRGNSAAVDARAASTRGGVSAFAFQGTNAHVAMARRGADAVSRSVDARWAAAEATRAWPAIAATRGVAPVRALPTPTRLRLVFQEEAESLERRAPAHALHVVVAAVRAALDERGTAASSGIRDAVVRSNAGWGAGDATAARAVDVDAARGAIEMTALVGGSSASVRVRARVATIVVVRTRSGDDFGVSDAEAAVSGLFPGSLSAHPPLSIPVLDAFQRQLTPFNSTPISSLRIERPSRRRNDAARAVADATALAAALDAAALALDHHLLGGSSNAGRLELAAASGCVVTSSADAFESSAEEAPMTTTTTGVSSSSAGAVVASVFILSRRRGDGGVVAELCGGVYNYARVGRTSPHASVGGGSREEEPPPRTPLPTYEVTWQATPERRARRPRCGFGSGFGGGGGAARAARVGRVRCAASALTALHARSPPDDDGGGDVRVVVAPSEWCLSGIVRAAEAESRKESGSSVSSSRALGGSITIATTSPSSSSSSSTRERGLTDRATDETGVEGAFSVSSLGAVARPKLLPRPRPTRAAAPPRVSSIAIVGGHGALASAVTTSLLQGGGGGGVRVVELYSRAGRPSSDDEPGHLANARSRRRGGWRCVFTAQSFDGGSRESVDARFGLNNDRCNGSGSGVARPVPGIVLRANGALRDALVPRQTAGTVRSIFASKASIGSVFLSTMHPLTADVLFSSVSALLGSPGQANYAAANAALDAAAAAAREEGRAVRSAQFGAWNTRDAGMAATAKRSNTSRYAAASSSTLARLERLGVGALEPEVGVAALWSILGEGGDDAPPATTAVTPFEWATFFGAHPSLAKEHLFSEYTGVIRHVSSSARGEESGIVGDDYISSCEDAKATRVADVGAAVAAAVAAVLGAAVDENEPLMAAGLDSLGVTELRAALRKDVGVEIPATALFDHPSVAALASFIGGEVVTRAMKASPSKPSKPGKPVLSTSSALAVDPSRRRLTPTTTIVGHACDTPTSSRARDGTRKMGKTGGGGGGGGATSSSSPLPMRFGAFLLRRVDRFDPDAFGAHRGEAKHVDPQQRLLLESTLAALLGAGDGDFVSVVGNPGGVAVAVGIQHMEYAHLGSGGGGGGGGGDRTGGDASPYAATGSALSVAAGRIAFAFGFGSTAVSVDTACSAALTAVHVARSGALHRGGFADDSSRLSNLNLNSYSNSERSASSASSSRHSVCGGANLMLGATNAAAIRAAGMLAVDGRCKTFDAGADGYGRGEACGVFLLRHSGPGGESDSDQKVPIGVLGVRVDASAANQDGRSGSLTAPNGLAQRAAIAEAWRESAPASFPRAVSTHGTGTALGDPIEMSALRAACGEIAATCTDSMERTADMEPLPATAAAASKSSVGHAEAAAGTTAILEVVTTLRARTIAPLKHLRTLNAHLSAALESHRVCTGNGDSGCIFIMPRGGGGAAACGAAGVSAFAFMGTNVHVVLSGDDDWSTSAPSSAPSREKTTLPWRRERSWRLDGPDGGVLALARVAATRVSRLAARMASSPAENGPEWRPLDGSSDRPFLVRADALAIAIDAATTAVFSGSSTGRFNGNALESTVSLTRATFLGGGGDPGWRGGGFAVDVDGVDGGVGVFAPSSRAIVMRSRVGRVLDVATANAAGFALGSFDGGAASIILEARRSTRVETYARAGSARGTRDAGRVAPLAIDAIVATLERGAYDIATVKSFSSAAADDDDADARRAVSLVASTDGAFVGGVGFGSSVRGTLGIRGTAASAGPDDGDYAREPTSSRATTKMDDVPKASSPKVAASDVAAVVAAVAADLAGGVAVSVDAPLLDAGIDSIAAVEFVTTVQSAVGVPLDVSEVATLPTMGAIVERVTNAVAAAAAAAAADEISDEISARRPGTSPAVAAVARDLSVADDGMIKSLKPARKPPPALFLGAPAFGDGPLAYMKLVHALPLGDHPVMTLERDTTSTPWPEAARTHASRIATSQPDGIIAIGGHSLGGLLAVESAVCLETEHGREVSCFLFDAPHPVQFKSEWNDVPGGGANSEEEEEGANSNSSSDDEEEESTGLAYMEVVLTSFHFDTIAAGWSGMTRDEKYATFEDVTFQATGRVVDARAMDEEISAGPYAAQWNSGIVRNEADGTCDVKAWKMLRGNADDEDDDDGGDGEKKNTKIFHRVRGKVTVYKASDESSALFETDLQMEHGGAVLRSVSGYAWALACDHLEIVHCQGSHMNLMTPEEDGGDLTHTIAPHLTRELSRAWGDVVVKPQTASNEALPTTTTAATTTTAPIVTLNDAERALETHGELGYDFAPWVAETWHDMRDADAASGAFYTLVPIRPRWRGGRRSLRSFAGASLRPPLAFNPRPRRLSTPTDAFELHPDIRLYRTALRRPRESRQARTSVLDASRGGRRRRPREPRGEVTRAGRVVRRRRARIKRPIARVARRREGRGRGRGRASGAFYTLVPIRPRSRGERRSLRTFAVNRRDATRRRDTVMVLVQDLVSDVEHWSAIALTARVPVIGVHLPSGLIPCSNDDDGDSGGDCGGGGGGNENENENDDDDARFAAVVVGATRAALNLHDRVENAEDAAVTTTTKKIIFAALPGTSAARVAFHAAMHFELCGIADACAVVALDGGAFYLTLVPIRPRSRGERRSLRTFAVVYFRPPLAFNPRPRRLSTPLLTPFNSTPISSLRMERPLDRDDVFSARLSKLPPRALDPAYQALAGKLQETETPGGWRAFRGF
